# The cost-effectiveness of using pneumococcal conjugate vaccine (PCV13) versus pneumococcal polysaccharide vaccine (PPSV23), in South African adults

**DOI:** 10.1371/journal.pone.0227945

**Published:** 2020-01-29

**Authors:** Charles Feldman, Sipho K. Dlamini, Shabir A. Madhi, Susan Meiring, Anne von Gottberg, Janetta C. de Beer, Margreet de Necker, Marthinus P. Stander

**Affiliations:** 1 Faculty of Health Sciences, University of the Witwatersrand, Johannesburg, South Africa; 2 Department of Medicine, University of Cape Town, Cape Town, South Africa; 3 Medical Research Council: Respiratory and Meningeal Pathogens Research Unit, Faculty of Health Sciences, University of the Witwatersrand, Johannesburg, South Africa; 4 Department of Science and Technology/National Research Foundation: Vaccine Preventable Diseases Research Chair, Faculty of Health Sciences, University of the Witwatersrand, Johannesburg, South Africa; 5 Division of Public Health Surveillance and Response, National Institute for Communicable Diseases (NICD), a division of the National Health Laboratory Service, Cape Town, South Africa; 6 Centre for Respiratory Diseases and Meningitis (CRDM), National Institute for Communicable Diseases (NICD), a division of the National Health Laboratory Service, Johannesburg, South Africa; 7 School of Pathology, Faculty of Health Sciences, University of the Witwatersrand, Johannesburg, South Africa; 8 TCD Outcomes Research, TCD Global, Centurion, South Africa; URCEco Ile de France Hopital de l'Hotel Dieu, FRANCE

## Abstract

*Streptococcus pneumoniae* (pneumococcus) remains an important cause of morbidity and mortality. Pneumococcal vaccination is part of the South African pediatric public immunization program but the potential cost-effectiveness of such an intervention for adults is unknown. This study aimed to compare the cost-effectiveness of two widely used pneumococcal vaccines: pneumococcal conjugate vaccine (PCV13) and pneumococcal polysaccharide vaccine (PPSV23) in South African adults, 18 years and older. Four analyses were carried out in a) both the private and public health care sectors; and b) for the HIV-infected population alone and for the total mixed population (all HIV-infected and -uninfected people). A previously published global pharmacoeconomic model was adapted and populated to represent the South African adult population. The model utilized a Markov-type process to depict the lifetime clinical and economic outcomes of patients who acquire pneumococcal disease in 2015, from a societal perspective. Costs were sourced in South African rand and converted to US dollar (USD). The incremental cost divided by the incremental effectiveness (expressed as quality-adjusted life years gained) represented the incremental cost-effectiveness ratio for PCV13 compared to PPSV23. Results indicated that the use of PCV13 compared to PPSV23 is highly cost-effective in the public sector cohorts with incremental cost-effectiveness ratios of $771 (R11,106)/quality-adjusted life year and $956 (R13,773)/quality-adjusted life year for the HIV-infected and mixed populations, respectively. The private sector cohort showed similar highly cost-effective results for the mixed population (incremental cost-effectiveness ratio $626 (R9,013)/quality-adjusted life year) and the HIV-infected cohort (dominant). In sensitivity analysis, the model was sensitive to vaccine price and effectiveness. Probabilistic sensitivity analyses found predominantly cost-effective ICERs. From a societal perspective, these findings provide some guidance to policy makers for consideration and implementation of an immunization strategy for both the public and private sector and amongst different adult patient pools in South Africa.

## Introduction

*Streptococcus pneumoniae*, also known as the pneumococcus, remains an important cause of morbidity and mortality in both developed and developing countries [1]. According to the World Health Organization (WHO), the majority of deaths occur in sub-Saharan Africa and Asia [2]. While the pneumococcus can produce infections in healthy individuals, these infections are more common in very young patients (<2 years), in the elderly (≥65 years), and in patients with underlying comorbid and/or immunocompromising disorders [3]. According to the Centers for Disease Control and Prevention (CDC) [3], adults between the ages of 18 and 64 years are at increased risk if they have chronic illnesses such as chronic respiratory, cardiac, hepatic (including alcoholism) or renal diseases, or diabetes mellitus. Similarly, people with immunodeficiency (including human immunodeficiency virus/acquired immunodeficiency syndrome [HIV/AIDS], cancer, and anatomical or functional asplenia); patients with cochlear implants or cerebrospinal fluid leaks; and cigarette smokers, are also at increased risk. Sequelae associated with pneumococcal meningitis include, amongst others, hearing loss, seizures, hydrocephalus, spasticity/paresis, cranial nerve palsies and visual impairment [4].

Given the risk of pneumococcal disease and its sequelae, a preventative strategy should be considered. Part of this preventative strategy could include vaccination. The pneumococcal polysaccharide vaccines (PPSV23), containing 23 serotypes of pneumococci, first became available in South Africa in 1992 (Pneumovax® [MSD] in 1992 and Imovax Pneumo 23® [Aventis] in 1998). PPSV23 affords potential protection against the 23 serotypes included in that vaccine (1, 2, 3, 4, 5, 6B, 7F, 8, 9N, 9V, 10A, 11A, 12F, 14, 15B, 17F, 18C, 19A, 19F, 20, 22F, 23F, and 33F). It is indicated for persons over 2 years of age who are at increased risk of acquiring invasive pneumococcal disease and its complications [5]. The first pneumococcal conjugate vaccine (PCV) that was registered in South Africa was PCV7 in 2003. PCV13 was initially registered for pediatric use in 2010 and was approved for adults (≥18 years) by 2015. PCV13 provides potential protection against the 13 serotypes included in the vaccine (1, 3, 4, 5, 6A, 6B, 7F, 9V, 14, 18C, 19A, 19F and 23F). It is indicated for the prevention of pneumococcal disease (including pneumonia and invasive disease) [6]. Pneumococcal vaccination is part of the South African pediatric public immunization program [7]. Vaccination of children with PCV7 was introduced in South Africa in 2009 [8]. By 2011, the guideline was updated to include PCV13 in favor of PCV7 [8]. After implementation of PCV13, population-based active surveillance of invasive pneumococcal disease showed a reduction in the overall incidence of the disease by 55% (95% CI: 30%-71%) in children younger than 2 years [8]. By 2012, some sources indicated that the estimated pediatric coverage of three doses of PCV13 was 99% [8]. However, other more recent sources (2013/14) show that this is most likely 86% [9].

Further to these direct effects, indirect effects (herd protection) are observed when there is widespread vaccination of a population, due to the reduction in nasopharyngeal carriage of vaccine-related serotypes in vaccinated children [10]. Accordingly, widespread vaccination of children with PCV could provide protection to unvaccinated individuals in the community (including adults), reducing pneumococcal disease incidence even among unvaccinated individuals.

Data on the true incidence of pneumococcal infections in South Africa are uncertain [1], but given the extent of the HIV epidemic in South Africa, pneumococcal disease is a significant problem [1]. A study before the era of routine PCV vaccination of children, documented that the incidence of invasive pneumococcal disease (IPD, consisting of pneumococcal meningitis and bacteremia) was much higher in HIV-infected patients compared to HIV-uninfected patients (214 cases versus 6.5 cases per 100,000 population, respectively) [11]. Furthermore, comprehensive antiretroviral therapy (ART) rollout during the period of the study did not significantly change the incidence of IPD in HIV-infected adult patients despite a stable prevalence of HIV infection [11]. Conversely, in another South African study, where 89% of IPD cases over 5 years of age were HIV co-infected, an increased incidence of IPD with increasing age was reported in both HIV-uninfected and HIV-infected persons, with an additional peak in the HIV-infected group in the later childhood years. Moreover, 39% of HIV-uninfected persons with IPD had chronic conditions and 19% of HIV-infected persons had chronic conditions predisposing them to IPD [12]. In total, 93% of all the cases had a condition predisposing them to pneumococcal disease according to the US Advisory Committee on Immunization Practices (ACIP) list of conditions requiring pneumococcal vaccination [12].

The mortality from pneumococcal disease remains high. A South African surveillance study found that the case-fatality rate (CFR) for meningitis caused by *S*. *pneumoniae* was 55%, while that for bacteremia was 23%, among adolescents and adults (aged 15 and older) [13]. Amongst other risk factors, increasing age and HIV infection were associated with increased mortality in these patients [13].

The high morbidity and mortality resulting from pneumococcal infections indicate a need for reducing the burden of IPD in HIV-infected patients via means other than ART alone. Vaccination in adults and children, providing direct and indirect protection to the community, is one way of reducing this burden [13]. However, a study on the vaccination of HIV-infected Ugandan adults with PPSV23 did not find it to be effective in preventing disease caused by *S*. *pneumoniae* compared to placebo [14]. Nevertheless, the authors later reported that despite a persistent excess of all-cause pneumonia in these vaccine recipients in a 6-year follow-up, they had paradoxically a 16% lower overall mortality risk in the PPSV23 vaccinated group compared to the placebo group [15]. Conversely, a second study showed that PCV7 vaccination in mostly HIV-infected adults in Malawi was effective in prevention of recurrent IPD [16]. The Community-Acquired Pneumonia Immunization Trial in Adults (CAPiTA) study was a randomized, double-blind, placebo-controlled trial of 84,496 Dutch adults (≥65 years). The study evaluated the efficacy of the PCV13 vaccination in preventing vaccine-type strains of pneumococcal community-acquired pneumonia (CAP), non-invasive and non-bacteremic CAP, and IPD [17]. Results indicated that PCV13 could reduce CAP by 45.6%; non-invasive and non-bacteremic CAP by 45%; and IPD by 75%. This trial is the basis for the vaccine efficacy data used in this current study [17].

PCV13 and PPSV23 are indicated and used in adults in South Africa. PPSV23 is recommended in South Africa as potentially beneficial to any individual and very effective in young otherwise healthy individuals, targeted at high-risk groups when there are cost considerations [1]. There is currently no National Department of Health policy on adult pneumococcal vaccination in South Africa, although a national guideline is in development by expert clinicians representing the various national societies of interest in South Africa. The only national recommendation for both PPSV23 and PCV13 vaccination in adults in South Africa is a section on vaccination included in the recently updated management guideline for community-acquired pneumonia in adults in South Africa [18]. The recommendations largely follow the Centers for Disease Control and Prevention recommendations for pneumococcal vaccination in adults in the United States.

Given this, the question arose whether an adult vaccination strategy would be cost-effective in the South African setting. The South African health care market consists of two tiers, the public health care sector and the private health care sector [19]. The provision of health care services in South Africa is divided along socioeconomic lines, with only approximately 50% of the health expenditure coming from the government, but approximately 86% of services being provided by the public health care sector, in public health care facilities (clinics, hospitals). The private sector caters to a small population with medium to high levels of income, in private health care facilities. This sector consists of those covered by medical insurance provided by medical schemes and those paying out-of-pocket for health care services. In general, PPSV23 is available and reimbursed in both the private and public sectors. PCV13 is available in the private sector and whether it is reimbursed at all, or how it is reimbursed (for example from medical scheme savings or a special benefits package), depends on the individual medical scheme the person belongs to. PCV13 is not available in the public sector.

The objective of this study was therefore to estimate the cost-effectiveness of the use of PCV13 compared to PPSV23 in South African adults in both the private and public health care sector from a societal perspective. Due to the high prevalence of HIV in South Africa, and the association of HIV infection with the incidence of pneumococcal disease, the cost-effectiveness of targeted pneumococcal vaccination of HIV-infected individuals was also investigated. Four scenarios were, therefore, created: i) A mixed private health care sector population (consisting of HIV-infected and HIV-uninfected individuals–the complete private health care sector population in South Africa); ii) A mixed public health care sector population (consisting of HIV-infected and HIV-uninfected individuals–the complete public health care sector population in South Africa); iii) A model for the HIV-infected population in the private health care sector; and iv) A model for the HIV-infected population in the public health care sector.

## Materials and methods

### Model description

A previously published global pharmacoeconomic model [20] was adapted and populated to represent the South African population of adults aged 18 years and older. A probabilistic Markov-type model was developed in Microsoft® Excel to depict the lifetime clinical and economic outcomes of alternative strategies for adult vaccination against pneumococcal disease. The study included both the private and public health care sectors and distinguished between i) the mixed population cohort (including both HIV-infected and -uninfected patients), as well as ii) a cohort of HIV-infected patients. Accordingly, we report on four theoretical South African population cohorts (private sector, public sector, HIV-infected private sector, and HIV-infected public sector).

The characteristics of each cohort of patients were stratified by age and risk of pneumococcal infection. Patients were classified into three risk categories: low-, moderate- and high-risk of acquiring pneumococcal disease (Table 1).

**Table 1:** Risk categories for pneumococcal disease.

Risk of pneumococcal disease	Description
Low-risk	Immunocompetent individuals without chronic medical conditions.
Moderate-risk	Immunocompetent individuals with any of the following: • One or more chronic medical conditions • Smoking [21] • Alcoholism [22] • Cochlear implants [23] • HIV infection (CD4 >350 cells/μl and virally suppressed)
High-risk [25]	Immunocompromized individuals with any of the following: • HIV infection (CD4 ≤350 cells/μl) • Chronic renal failure • Cancer [24] • Solid organ transplant recipients [25]

The study’s cohort of patients then entered the model via two potential scenarios: vaccination with PPSV23 or vaccination with PCV13. The vaccination of patients was dependent on their age, risk profile, and vaccination history.

The vaccination strategy was based on supplier coverage calculations. For patients in the private sector mixed and HIV-infected models, vaccination was assumed to be performed only for high-risk patients in the 18–49 and 50-64-year age groups (40%). Overall, 30% of patients in the 65–74 age group (all risk groups) were considered to be vaccinated, while 20% of all patients aged 75–99 (all risk groups) were considered to be vaccinated. For the public sector mixed and HIV-infected models, vaccination was assumed to be performed only for high-risk patients in the 18–49 and 50-64-year age groups (1%). For subsequent age groups, 1% of all patients in each risk group were considered to be vaccinated.

As a model base case, in order to calculate the actual number of patients vaccinated based on the above vaccination strategy, the following assumptions were made: No revaccination after completion of the vaccine regime; the effectiveness of vaccines are linked to risk stratification of patients, i.e. vaccines are less effective in higher risk patients (for example, HIV-infected patients).

Expected outcomes were evaluated for each person in the model population on an annual basis, from model entry, through to the end of the modelling horizon. The modelling horizon was set to a lifetime horizon of 82 years and was modelled from a societal perspective, considering both direct medical and indirect costs. Indirect costs considered were loss of paid productivity due to the disease. Furthermore, in accordance with the South African published Pharmacoeconomic Guidelines [26], the discount rates of 5% per annum for both costs and benefits were used. In each year, pneumococcal-related outcomes were projected for each person in the model cohort based on age, risk profile, vaccination status, vaccine type (PPSV23 or PCV13), and time since vaccination.

**Fig 1:**
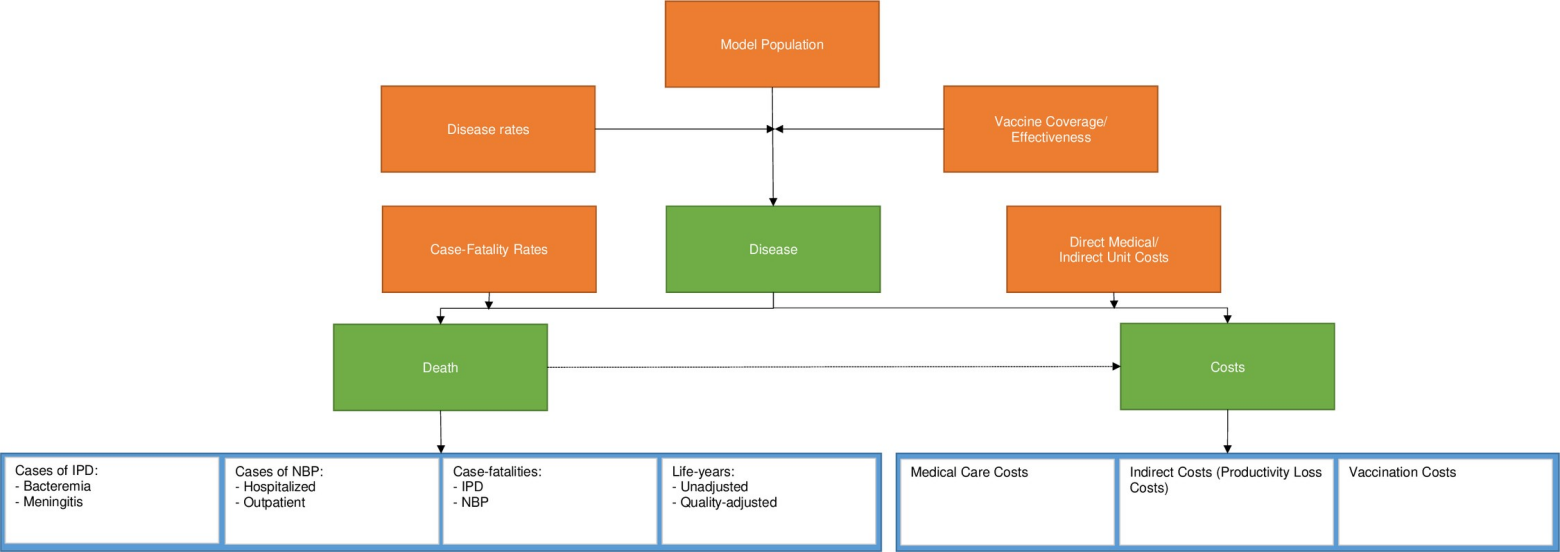
Model schematic. IPD, invasive pneumococcal disease; NBP, non-bacteremic pneumonia.

A schematic diagram of the model is shown in Fig 1. The model consists of several modules, including disease, death and costs, which impact on one another. The inputs required for the disease module include disease rates (incidence), model population, vaccine coverage and vaccine effectiveness. Inputs required for the death module include case-fatality rates. Inputs required for the cost module include direct medical and indirect unit costs, vaccine coverage and vaccine effectiveness. Outcomes include the number of IPD and non-bacteremic pneumonia (NBP) cases; case-fatalities for IPD and NBP; life years (unadjusted and quality-adjusted); medical care costs; indirect costs, and vaccination costs. Model health states included death, low-risk, medium-risk, high-risk, IPD, inpatient NBP and outpatient NBP. A schematic diagram for the Markov model, indicating the transitions between model health states, is shown in the supplementary material in [20].

Bacteremia in the study’s model included all cases of invasive pneumococcal disease, except for meningitis, which was handled separately in the model. Non-bacteremic pneumonia in the model included all cases of pneumonia caused by *S*. *pneumoniae*, which are not included under the definition of bacteremia.

A cost-effectiveness threshold, or willingness-to-pay (WTP) threshold, of approximately $13,889 (R200,000) was used and was based on three times the GDP per capita for South Africa, based on previous funding decisions in the private sector [27,28,29]. According to this threshold, a vaccine with incremental cost-effectiveness ratio (ICER) less than $13,889 (R200,000) per quality-adjusted life year (QALY) is considered as providing good value for money (cost-effective), whereas treatments with an ICER greater than $13,889 (R200,000) are typically viewed as a poor use of resources (not cost-effective) [30].

### Data inputs

The majority of data was sourced from the literature. However, where data points were not available in the literature or where there was uncertainty regarding some inputs, an advisory board consisting of eight key opinion leaders was set up to obtain consensus answers using a modified two round Delphi panel method [31].

For numeric responses provided by the Delphi panel, the coefficient of variation (CV) was calculated. The CV was used to determine whether consensus was achieved [32]:

- CV between 0 and 0.5—good consensus, no additional round.
- CV between 0.5 and 0.8—less than satisfactory degree of consensus, possible need for an additional round.
- CV greater than 0.8—poor degree of consensus, definite need for an additional round.

For dichotomous responses (Yes or No), consensus was achieved when 70% of the panelists answered Yes or No for sensitive variables and 60% of the panelists answered Yes or No for non-sensitive variables. If consensus was not achieved after the first round, a second round was undertaken. Before the second round, the first round’s results were discussed between the panelists. If no consensus was achieved after the second round, disclosure of this fact and sensitivity analysis on the parameter under question would be performed.

#### Clinical data

The population was categorized in five age bands (18–49, 50–64, 65–74, 75–84 and 85–99 years), and further stratified into three risk groups (low, moderate and high) as illustrated in Tables 2–5, which show the base case model inputs for each of the four models. The model allowed for patients to move between different risk groups as they aged in a dynamic way.

**Table 2:** Base case model inputs for the mixed private health care sector population.

	18–49 years			50–64 years			65–74 years			75–84 years			85–99 years		
Risk group	Low	Moderate	High	Low	Moderate	High	Low	Moderate	High	Low	Moderate	High	Low	Moderate	High
Population numbers	3,174,599	1,221,345	94,295	589,083	707,957	23,775	97,259	308,819	9,560	25,087	152,751	7,991	7,695	35,072	1,459
Percentage distribution of patients according to disease risk (%)	70.7	27.2	2.1	44.6	53.6	1.8	23.4	74.3	2.3	13.5	82.2	4.3	17.4	79.3	3.3
Annual incidence of disease (per 100,000)															
Bacteremia	10.3	89.3	458.5	6.2	53.6	275.4	1.5	12.6	64.5	1.2	10.5	54.1	1.3	11.3	58.2
Meningitis	3.2	28.1	144.1	1.9	16.9	86.5	0.5	3.9	20.3	0.4	3.3	17.0	0.4	3.6	18.3
Inpatient all-cause pneumonia	2.9	24.9	127.9	4.0	34.2	175.6	1.7	14.8	75.8	2.1	18.5	94.9	2.8	24.4	125.2
Outpatient all-cause pneumonia	25.9	224.3	1151.3	13.3	115.5	592.7	2.3	20.3	104.1	1.3	10.9	56.1	0.8	7.2	37.1
Annual mortality rate in general population (per 100)	0.6	0.6	2.1	1.6	1.6	5.9	2.9	2.9	10.9	8.9	8.9	33.8	6.8	6.8	25.7
Annual case-fatality rate (per 100)															
Bacteremia	19.1	19.1	32.3	33.2	33.2	56.1	39.2	39.2	66.3	38.7	38.7	65.4	39.0	39.0	65.9
Meningitis	51.4	51.4	86.9	63.4	63.4	100.0	73.8	73.8	100.0	72.8	72.8	100.0	73.3	73.3	100.0
Inpatient all-cause pneumonia	5.0	5.0	8.4	8.7	8.7	14.7	10.3	10.3	17.3	10.1	10.1	17.1	10.2	10.2	17.2
Outpatient all-cause pneumonia	0.0	0.0	0.0	0.0	0.0	0.0	0.0	0.0	0.0	0.0	0.0	0.0	0.0	0.0	0.0
Utilities															
Health-state utility for general population	0.8996	0.8996	0.8245	0.8101	0.8101	0.6897	0.7542	0.7542	0.5688	0.6792	0.6792	0.5206	0.5280	0.5280	0.5071
Disutility due to bacteremia	0.0079	0.0079	0.0079	0.0079	0.0079	0.0079	0.0079	0.0079	0.0079	0.0079	0.0079	0.0079	0.0079	0.0079	0.0079
Disutility due to meningitis	0.0232	0.0232	0.0232	0.0232	0.0232	0.0232	0.0232	0.0232	0.0232	0.0232	0.0232	0.0232	0.0232	0.0232	0.0232
Disutility due to inpatient all-cause pneumonia	0.0060	0.0060	0.0060	0.0060	0.0060	0.0060	0.0060	0.0060	0.0060	0.0060	0.0060	0.0060	0.0060	0.0060	0.0060
Disutility due to outpatient all-cause pneumonia	0.0040	0.0040	0.0040	0.0040	0.0040	0.0040	0.0040	0.0040	0.0040	0.0040	0.0040	0.0040	0.0040	0.0040	0.0040
Effectiveness of PPSV23 against vaccine-type IPD (%)															
Year 0	93.0	93.0	21.0	87.3	87.3	13.7	76.6	76.6	1.4	67.8	67.8	0.0	59.4	59.4	0.0
Year 5	77.1	77.1	18.6	69.0	69.0	12.2	54.1	54.1	0.5	41.3	41.3	0.0	28.8	28.8	0.0
Year 10	29.3	29.3	8.9	22.8	22.8	5.8	12.3	12.3	0.2	4.5	4.5	0.0	0.0	0.0	0.0
Year 15	4.1	4.1	1.8	2.7	2.7	1.2	0.9	0.9	0.0	0.1	0.1	0.0	0.0	0.0	0.0
Year 16	1.0	1.0	0.3	0.7	0.7	0.2	0.2	0.2	0.0	0.0	0.0	0.0	0.0	0.0	0.0
Effectiveness of PCV13 against vaccine-type IPD (%)															
Year 0	84.6	84.6	66.0	82.0	82.0	63.9	76.8	76.8	59.9	72.2	72.2	56.4	67.6	67.6	52.7
Year 5	84.1	84.1	65.6	80.9	80.9	63.1	74.5	74.5	58.1	68.5	68.5	53.5	61.5	61.5	48.0
Year 10	59.6	59.6	46.5	53.3	53.3	41.6	41.0	41.0	32.0	27.7	27.7	21.6	1.1	1.1	0.8
Year 15	25.1	25.1	19.6	21.0	21.0	16.4	13.8	13.8	10.8	4.5	4.5	3.5	0.0	0.0	0.0
Year 16	23.0	23.0	18.0	19.3	19.3	15.0	12.7	12.7	9.9	4.1	4.1	3.2	0.0	0.0	0.0
Effectiveness of PPSV23 against inpatient all-cause pneumonia (%)															
Year 0	0.0	0.0	0.0	0.0	0.0	0.0	0.0	0.0	0.0	0.0	0.0	0.0	0.0	0.0	0.0
Year 5	0.0	0.0	0.0	0.0	0.0	0.0	0.0	0.0	0.0	0.0	0.0	0.0	0.0	0.0	0.0
Year 10	0.0	0.0	0.0	0.0	0.0	0.0	0.0	0.0	0.0	0.0	0.0	0.0	0.0	0.0	0.0
Year 15	0.0	0.0	0.0	0.0	0.0	0.0	0.0	0.0	0.0	0.0	0.0	0.0	0.0	0.0	0.0
Year 16	0.0	0.0	0.0	0.0	0.0	0.0	0.0	0.0	0.0	0.0	0.0	0.0	0.0	0.0	0.0
Effectiveness of PCV13 against inpatient all-cause pneumonia (%)															
Year 0	21.6	21.6	14.0	20.9	20.9	13.6	19.6	19.6	12.7	18.4	18.4	12.0	17.2	17.2	11.2
Year 5	21.4	21.4	13.9	20.6	20.6	13.4	19.0	19.0	12.4	17.5	17.5	11.4	15.7	15.7	10.2
Year 10	15.2	15.2	9.9	13.6	13.6	8.8	10.5	10.5	6.8	7.1	7.1	4.6	0.3	0.3	0.2
Year 15	6.4	6.4	4.2	5.4	5.4	3.5	3.5	3.5	2.3	1.1	1.1	0.7	0.0	0.0	0.0
Year 16	5.9	5.9	3.8	4.9	4.9	3.2	3.2	3.2	2.1	1.0	1.0	0.7	0.0	0.0	0.0
Effectiveness of PPSV23 against outpatient all-cause pneumonia (%)															
Year 0	0.0	0.0	0.0	0.0	0.0	0.0	0.0	0.0	0.0	0.0	0.0	0.0	0.0	0.0	0.0
Year 5	0.0	0.0	0.0	0.0	0.0	0.0	0.0	0.0	0.0	0.0	0.0	0.0	0.0	0.0	0.0
Year 10	0.0	0.0	0.0	0.0	0.0	0.0	0.0	0.0	0.0	0.0	0.0	0.0	0.0	0.0	0.0
Year 15	0.0	0.0	0.0	0.0	0.0	0.0	0.0	0.0	0.0	0.0	0.0	0.0	0.0	0.0	0.0
Year 16	0.0	0.0	0.0	0.0	0.0	0.0	0.0	0.0	0.0	0.0	0.0	0.0	0.0	0.0	0.0
Effectiveness of PCV13 against inpatient all-cause pneumonia (%)															
Year 0	21.6	21.6	14.0	20.9	20.9	13.6	19.6	19.6	12.7	18.4	18.4	12.0	17.2	17.2	11.2
Year 5	21.4	21.4	13.9	20.6	20.6	13.4	19.0	19.0	12.4	17.5	17.5	11.4	15.7	15.7	10.2
Year 10	15.2	15.2	9.9	13.6	13.6	8.8	10.5	10.5	6.8	7.1	7.1	4.6	0.3	0.3	0.2
Year 15	6.4	6.4	4.2	5.4	5.4	3.5	3.5	3.5	2.3	1.1	1.1	0.7	0.0	0.0	0.0
Year 16	5.9	5.9	3.8	4.9	4.9	3.2	3.2	3.2	2.1	1.0	1.0	0.7	0.0	0.0	0.0
Medical care costs (per case)															
Bacteremia	$1,886 (R27,156)	$1,886 (R27,156)	$2,219 (R31,948)	$1,932 (R27,827)	$1,932 (R27,827)	$2,265 (R32,619)	$2,076 (R29,887)	$2,505 (R36,069)	$2,838 (R40,862)	$2,551 (R36,740)	$2,788 (R40,143)	$3,027 (R43,594)	$2,884 (R41,533)	$2,884 (R41,533)	$3,264 (R46,996)
Meningitis	$5,073 (R73,057)	$5,073 (R73,057)	$5,250 (R75,597)	$5,073 (R73,057)	$5,073 (R73,057)	$5,250 (R75,597)	$5,191 (R74,750)	$5,191 (R74,750)	$5,372 (R77,351)	$5,493 (R79,104)	$5,493 (R79,104)	$5,733 (R82,551)	$5,733 (R82,551)	$5,733 (R82,551)	$5,733 (R82,551)
Inpatient all-cause pneumonia	$1,642 (R23,642)	$1,738 (R25,032)	$2,025 (R29,153)	$1,642 (R23,642)	$1,881 (R27,092)	$2,071 (R29,824)	$2,214 (R31,885)	$2,357 (R33,946)	$2,690 (R38,738)	$2,640 (R38,019)	$2,926 (R42,141)	$3,402 (R48,994)	$3,116 (R44,872)	$3,116 (R44,872)	$3,592 (R51,725)
Outpatient all-cause pneumonia	$53 (R767)	$53 (R767)	$53 (R767)	$53 (R767)	$53 (R767)	$53 (R767)	$53 (R767)	$53 (R767)	$53 (R767)	$53 (R767)	$53 (R767)	$53 (R767)	$53 (R767)	$53 (R767)	$53 (R767)
Vaccination costs (per vaccination)															
PPSV23	$9.22 (R132.83)	$9.22 (R132.83)	$9.22 (R132.83)	$9.22 (R132.83)	$9.22 (R132.83)	$9.22 (R132.83)	$9.22 (R132.83)	$9.22 (R132.83)	$9.22 (R132.83)	$9.22 (R132.83)	$9.22 (R132.83)	$9.22 (R132.83)	$9.22 (R132.83)	$9.22 (R132.83)	$9.22 (R132.83)
PCV13	$47.59 (R685.27)	$47.59 (R685.27)	$47.59 (R685.27)	$47.59 (R685.27)	$47.59 (R685.27)	$47.59 (R685.27)	$47.59 (R685.27)	$47.59 (R685.27)	$47.59 (R685.27)	$47.59 (R685.27)	$47.59 (R685.27)	$47.59 (R685.27)	$47.59 (R685.27)	$47.59 (R685.27)	$47.59 (R685.27)
Administration costs for PPSV23	$24.96 (R359.42)	$24.96 (R359.42)	$24.96 (R359.42)	$24.96 (R359.42)	$24.96 (R359.42)	$24.96 (R359.42)	$24.96 (R359.42)	$24.96 (R359.42)	$24.96 (R359.42)	$24.96 (R359.42)	$24.96 (R359.42)	$24.96 (R359.42)	$24.96 (R359.42)	$24.96 (R359.42)	$24.96 (R359.42)
Administration costs for PCV13	$2.58 (R37.13)	$2.58 (R37.13)	$2.58 (R37.13)	$2.58 (R37.13)	$2.58 (R37.13)	$2.58 (R37.13)	$2.58 (R37.13)	$2.58 (R37.13)	$2.58 (R37.13)	$2.58 (R37.13)	$2.58 (R37.13)	$2.58 (R37.13)	$2.58 (R37.13)	$2.58 (R37.13)	$2.58 (R37.13)

**Table 3:** Base case model inputs for the mixed public health care sector population.

	18–49 years			50–64 years			65–74 years			75–84 years			85–99 years		
Risk group	Low	Moderate	High	Low	Moderate	Risk group	Low	Moderate	High	Low	Moderate	Risk group	Low	Moderate	High
Population numbers	17,263,356	4,077,766	3,076,638	2,061,182	2,167,476	392,826	372,549	1,165,411	54,131	46,581	285,010	13,457	67,695	316,690	4,669
Percentage distribution of patients according to disease risk (%)	70.7	16.7	12.6	44.6	46.9	8.5	23.4	73.2	3.4	13.5	82.6	3.9	17.4	81.4	1.2
Annual incidence of disease (per 100,000)															
Bacteremia	5.3	46.2	237.3	4.4	38.1	195.7	1.4	12.0	61.5	1.2	10.7	55.0	1.4	12.4	63.9
Meningitis	1.7	14.5	74.6	1.4	12.0	61.5	0.4	3.8	19.3	0.4	3.4	17.3	0.5	3.9	20.1
Inpatient all-cause pneumonia	9.7	83.8	430.3	8.6	74.5	382.2	3.0	26.3	134.8	3.0	26.0	133.7	3.6	31.3	160.5
Outpatient all-cause pneumonia	5.2	45.1	231.7	3.7	31.9	163.8	0.8	7.2	36.8	0.4	3.8	19.7	0.4	3.5	17.8
Annual mortality rate in general population (per 100)	0.4	0.4	1.7	1.3	1.3	5.0	2.8	2.8	10.6	9.0	9.0	34.2	7.1	7.1	27.2
Annual case-fatality rate (per 100)															
Bacteremia	17.8	17.8	30.1	31.7	31.7	53.6	39.0	39.0	65.9	38.8	38.8	65.6	39.5	39.5	66.8
Meningitis	48.0	48.0	81.1	60.7	60.7	100.0	73.3	73.3	100.0	73.0	73.0	100.0	74.4	74.4	100.0
Inpatient all-cause pneumonia	4.7	4.7	7.9	8.3	8.3	14.0	10.2	10.2	17.2	10.2	10.2	17.2	10.3	10.3	17.5
Outpatient all-cause pneumonia	0.0	0.0	0.0	0.0	0.0	0.0	0.0	0.0	0.0	0.0	0.0	0.0	0.0	0.0	0.0
Utilities															
Health-state utility for general population	0.8996	0.8996	0.8245	0.8101	0.8101	0.6897	0.7542	0.7542	0.5688	0.6792	0.6792	0.5206	0.5280	0.5280	0.5071
Disutility due to bacteremia	0.0079	0.0079	0.0079	0.0079	0.0079	0.0079	0.0079	0.0079	0.0079	0.0079	0.0079	0.0079	0.0079	0.0079	0.0079
Disutility due to meningitis	0.0232	0.0232	0.0232	0.0232	0.0232	0.0232	0.0232	0.0232	0.0232	0.0232	0.0232	0.0232	0.0232	0.0232	0.0232
Disutility due to inpatient all-cause pneumonia	0.0060	0.0060	0.0060	0.0060	0.0060	0.0060	0.0060	0.0060	0.0060	0.0060	0.0060	0.0060	0.0060	0.0060	0.0060
Disutility due to outpatient all-cause pneumonia	0.0040	0.0040	0.0040	0.0040	0.0040	0.0040	0.0040	0.0040	0.0040	0.0040	0.0040	0.0040	0.0040	0.0040	0.0040
Effectiveness of PPSV23 against vaccine-type IPD (%)															
Year 0	93.0	93.0	21.0	87.3	87.3	13.7	76.6	76.6	1.4	67.8	67.8	0.0	59.4	59.4	0.0
Year 5	77.1	77.1	18.6	69.0	69.0	12.2	54.1	54.1	0.5	41.3	41.3	0.0	28.8	28.8	0.0
Year 10	29.3	29.3	8.9	22.8	22.8	5.8	12.3	12.3	0.2	4.5	4.5	0.0	0.0	0.0	0.0
Year 15	4.1	4.1	1.8	2.7	2.7	1.2	0.9	0.9	0.0	0.1	0.1	0.0	0.0	0.0	0.0
Year 16	1.0	1.0	0.3	0.7	0.7	0.2	0.2	0.2	0.0	0.0	0.0	0.0	0.0	0.0	0.0
Effectiveness of PCV13 against vaccine-type IPD (%)															
Year 0	84.6	84.6	66.0	82.0	82.0	63.9	76.8	76.8	59.9	72.2	72.2	56.4	67.6	67.6	52.7
Year 5	84.1	84.1	65.6	80.9	80.9	63.1	74.5	74.5	58.1	68.5	68.5	53.5	61.5	61.5	48.0
Year 10	59.6	59.6	46.5	53.3	53.3	41.6	41.0	41.0	32.0	27.7	27.7	21.6	1.1	1.1	0.8
Year 15	25.1	25.1	19.6	21.0	21.0	16.4	13.8	13.8	10.8	4.5	4.5	3.5	0.0	0.0	0.0
Year 16	23.0	23.0	18.0	19.3	19.3	15.0	12.7	12.7	9.9	4.1	4.1	3.2	0.0	0.0	0.0
Effectiveness of PPSV23 against inpatient all-cause pneumonia (%)															
Year 0	0.0	0.0	0.0	0.0	0.0	0.0	0.0	0.0	0.0	0.0	0.0	0.0	0.0	0.0	0.0
Year 5	0.0	0.0	0.0	0.0	0.0	0.0	0.0	0.0	0.0	0.0	0.0	0.0	0.0	0.0	0.0
Year 10	0.0	0.0	0.0	0.0	0.0	0.0	0.0	0.0	0.0	0.0	0.0	0.0	0.0	0.0	0.0
Year 15	0.0	0.0	0.0	0.0	0.0	0.0	0.0	0.0	0.0	0.0	0.0	0.0	0.0	0.0	0.0
Year 16	0.0	0.0	0.0	0.0	0.0	0.0	0.0	0.0	0.0	0.0	0.0	0.0	0.0	0.0	0.0
Effectiveness of PCV13 against inpatient all-cause pneumonia (%)															
Year 0	21.6	21.6	14.0	20.9	20.9	13.6	19.6	19.6	12.7	18.4	18.4	12.0	17.2	17.2	11.2
Year 5	21.4	21.4	13.9	20.6	20.6	13.4	19.0	19.0	12.4	17.5	17.5	11.4	15.7	15.7	10.2
Year 10	15.2	15.2	9.9	13.6	13.6	8.8	10.5	10.5	6.8	7.1	7.1	4.6	0.3	0.3	0.2
Year 15	6.4	6.4	4.2	5.4	5.4	3.5	3.5	3.5	2.3	1.1	1.1	0.7	0.0	0.0	0.0
Year 16	5.9	5.9	3.8	4.9	4.9	3.2	3.2	3.2	2.1	1.0	1.0	0.7	0.0	0.0	0.0
Effectiveness of PPSV23 against outpatient all-cause pneumonia (%)															
Year 0	0.0	0.0	0.0	0.0	0.0	0.0	0.0	0.0	0.0	0.0	0.0	0.0	0.0	0.0	0.0
Year 5	0.0	0.0	0.0	0.0	0.0	0.0	0.0	0.0	0.0	0.0	0.0	0.0	0.0	0.0	0.0
Year 10	0.0	0.0	0.0	0.0	0.0	0.0	0.0	0.0	0.0	0.0	0.0	0.0	0.0	0.0	0.0
Year 15	0.0	0.0	0.0	0.0	0.0	0.0	0.0	0.0	0.0	0.0	0.0	0.0	0.0	0.0	0.0
Year 16	0.0	0.0	0.0	0.0	0.0	0.0	0.0	0.0	0.0	0.0	0.0	0.0	0.0	0.0	0.0
Effectiveness of PCV13 against inpatient all-cause pneumonia (%)															
Year 0	21.6	21.6	14.0	20.9	20.9	13.6	19.6	19.6	12.7	18.4	18.4	12.0	17.2	17.2	11.2
Year 5	21.4	21.4	13.9	20.6	20.6	13.4	19.0	19.0	12.4	17.5	17.5	11.4	15.7	15.7	10.2
Year 10	15.2	15.2	9.9	13.6	13.6	8.8	10.5	10.5	6.8	7.1	7.1	4.6	0.3	0.3	0.2
Year 15	6.4	6.4	4.2	5.4	5.4	3.5	3.5	3.5	2.3	1.1	1.1	0.7	0.0	0.0	0.0
Year 16	5.9	5.9	3.8	4.9	4.9	3.2	3.2	3.2	2.1	1.0	1.0	0.7	0.0	0.0	0.0
Medical care costs (per case)															
Bacteremia	$1,152 (R16,589)	$1,176 (R16,931)	$1,267 (R18,245)	$1,176 (R16,931)	$1,198 (R17,246)	$1,267 (R18,245)	$1,267 (R18,245)	$1,335 (R19,217)	$1,450 (R20,873)	$1,471 (R21,188)	$1,517 (R21,845)	$1,632 (R23,501)	$1,517 (R21,845)	$1,563 (R22,502)	$1,678 (R24,158)
Meningitis	$3,911 (R56,323)	$3,911 (R56,323)	$4,193 (R60,382)	$3,869 (R55,717)	$3,869 (R55,717)	$4,154 (R59,822)	$3,788 (R54,551)	$3,788 (R54,551)	$3,911 (R56,323)	$3,788 (R54,551)	$3,788 (R54,551)	$3,911 (R56,323)	$3,911 (R56,323)	$3,911 (R56,323)	$4,073 (R58,656)
Inpatient all-cause pneumonia	$1,025 (R14,765)	$1,071 (R15,422)	$1,139 (R16,395)	$1,139 (R16,395)	$1,208 (R17,393)	$1,321 (R19,023)	$1,321 (R19,023)	$1,345 (R19,364)	$1,458 (R20,994)	$1,504 (R21,651)	$1,527 (R21,992)	$1,595 (R22,965)	$1,755 (R25,277)	$1,755 (R25,277)	$1,847 (R26,591)
Outpatient all-cause pneumonia	$24 (R348)	$24 (R348)	$24 (R348)	$24 (R348)	$24 (R348)	$24 (R348)	$24 (R348)	$24 (R348)	$24 (R348)	$24 (R348)	$24 (R348)	$24 (R348)	$24 (R348)	$24 (R348)	$24 (R348)
Vaccination costs (per vaccination)															
PPSV23	$7.07 (R101.85)	$7.07 (R101.85)	$7.07 (R101.85)	$7.07 (R101.85)	$7.07 (R101.85)	$7.07 (R101.85)	$7.07 (R101.85)	$7.07 (R101.85)	$7.07 (R101.85)	$7.07 (R101.85)	$7.07 (R101.85)	$7.07 (R101.85)	$7.07 (R101.85)	$7.07 (R101.85)	$7.07 (R101.85)
PCV13	$47.59 (R685.27)	$47.59 (R685.27)	$47.59 (R685.27)	$47.59 (R685.27)	$47.59 (R685.27)	$47.59 (R685.27)	$47.59 (R685.27)	$47.59 (R685.27)	$47.59 (R685.27)	$47.59 (R685.27)	$47.59 (R685.27)	$47.59 (R685.27)	$47.59 (R685.27)	$47.59 (R685.27)	$47.59 (R685.27)
Administration costs for PPSV23	$18.67 (R268.85)	$18.67 (R268.85)	$18.67 (R268.85)	$18.67 (R268.85)	$18.67 (R268.85)	$18.67 (R268.85)	$18.67 (R268.85)	$18.67 (R268.85)	$18.67 (R268.85)	$18.67 (R268.85)	$18.67 (R268.85)	$18.67 (R268.85)	$18.67 (R268.85)	$18.67 (R268.85)	$18.67 (R268.85)
Administration costs for PCV13	$0.00 (R0.00)	$0.00 (R0.00)	$0.00 (R0.00)	$0.00 (R0.00)	$0.00 (R0.00)	$0.00 (R0.00)	$0.00 (R0.00)	$0.00 (R0.00)	$0.00 (R0.00)	$0.00 (R0.00)	$0.00 (R0.00)	$0.00 (R0.00)	$0.00 (R0.00)	$0.00 (R0.00)	$0.00 (R0.00)

**Table 4:** Base case model inputs for the HIV-infected private health care sector population.

	18–49 years			50–64 years			65–74 years			75–84 years			85–99 years		
Risk group	Low	Moderate	High	Low	Moderate	Risk group	Low	Moderate	High	Low	Moderate	Risk group	Low	Moderate	High
Population numbers	0	49,921	87,602	0	5,746	10,083	0	414	726	0	8	14	0	0	0
Percentage distribution of patients according to disease risk (%)	0	36.3	63.7	0	36.3	63.7	0	36.3	63.7	0	36.3	63.7	0	36.3	63.7
Annual incidence of disease (per 100,000)															
Bacteremia	128.7	128.7	128.7	213.1	213.1	213.1	637.1	637.1	637.1	637.1	637.1	637.1	637.1	637.1	637.1
Meningitis	39.9	39.9	39.9	66.2	66.2	66.2	197.8	197.8	197.8	197.8	197.8	197.8	197.8	197.8	197.8
Inpatient all-cause pneumonia	35.9	35.9	35.9	135.9	135.9	135.9	749.1	749.1	749.1	1117.4	1117.4	1117.4	1371.2	1371.2	1371.2
Outpatient all-cause pneumonia	323.0	323.0	323.0	458.7	458.7	458.7	1028.5	1028.5	1028.5	660.2	660.2	660.2	406.4	406.4	406.4
Annual mortality rate in general population (per 100)	0.6	2.3	2.3	1.6	6.2	6.2	3.1	11.6	11.6	10.0	37.9	37.9	7.4	28.1	28.1
Annual case-fatality rate (per 100)															
Bacteremia	19.4	32.7	32.7	33.6	56.8	56.8	39.9	67.4	67.4	39.9	67.4	67.4	39.9	67.4	67.4
Meningitis	52.1	88.1	88.1	64.2	100.0	100.0	75.0	100.0	100.0	75.0	100.0	100.0	75.0	100.0	100.0
Inpatient all-cause pneumonia	8.6	14.5	14.5	14.8	25.1	25.1	17.6	29.8	29.8	17.6	29.8	29.8	17.6	29.8	29.8
Outpatient all-cause pneumonia	0.0	0.0	0.0	0.0	0.0	0.0	0.0	0.0	0.0	0.0	0.0	0.0	0.0	0.0	0.0
Utilities															
Health-state utility for general population	0.8996	0.8996	0.8245	0.8101	0.8101	0.6897	0.7542	0.7542	0.5688	0.6792	0.6792	0.5206	0.5280	0.5280	0.5071
Disutility due to bacteremia	0.0079	0.0079	0.0079	0.0079	0.0079	0.0079	0.0079	0.0079	0.0079	0.0079	0.0079	0.0079	0.0079	0.0079	0.0079
Disutility due to meningitis	0.0232	0.0232	0.0232	0.0232	0.0232	0.0232	0.0232	0.0232	0.0232	0.0232	0.0232	0.0232	0.0232	0.0232	0.0232
Disutility due to inpatient all-cause pneumonia	0.0060	0.0060	0.0060	0.0060	0.0060	0.0060	0.0060	0.0060	0.0060	0.0060	0.0060	0.0060	0.0060	0.0060	0.0060
Disutility due to outpatient all-cause pneumonia	0.0040	0.0040	0.0040	0.0040	0.0040	0.0040	0.0040	0.0040	0.0040	0.0040	0.0040	0.0040	0.0040	0.0040	0.0040
Effectiveness of PPSV23 against vaccine-type IPD (%)															
Year 0	93.0	93.0	21.0	87.3	87.3	13.7	76.6	76.6	1.4	67.8	67.8	0.0	59.4	59.4	0.0
Year 5	77.1	77.1	18.6	69.0	69.0	12.2	54.1	54.1	0.5	41.3	41.3	0.0	28.8	28.8	0.0
Year 10	29.3	29.3	8.9	22.8	22.8	5.8	12.3	12.3	0.2	4.5	4.5	0.0	0.0	0.0	0.0
Year 15	4.1	4.1	1.8	2.7	2.7	1.2	0.9	0.9	0.0	0.1	0.1	0.0	0.0	0.0	0.0
Year 16	1.0	1.0	0.3	0.7	0.7	0.2	0.2	0.2	0.0	0.0	0.0	0.0	0.0	0.0	0.0
Effectiveness of PCV13 against vaccine-type IPD (%)															
Year 0	84.6	84.6	66.0	82.0	82.0	63.9	76.8	76.8	59.9	72.2	72.2	56.4	67.6	67.6	52.7
Year 5	84.1	84.1	65.6	80.9	80.9	63.1	74.5	74.5	58.1	68.5	68.5	53.5	61.5	61.5	48.0
Year 10	59.6	59.6	46.5	53.3	53.3	41.6	41.0	41.0	32.0	27.7	27.7	21.6	1.1	1.1	0.8
Year 15	25.1	25.1	19.6	21.0	21.0	16.4	13.8	13.8	10.8	4.5	4.5	3.5	0.0	0.0	0.0
Year 16	23.0	23.0	18.0	19.3	19.3	15.0	12.7	12.7	9.9	4.1	4.1	3.2	0.0	0.0	0.0
Effectiveness of PPSV23 against inpatient all-cause pneumonia (%)															
Year 0	0.0	0.0	0.0	0.0	0.0	0.0	0.0	0.0	0.0	0.0	0.0	0.0	0.0	0.0	0.0
Year 5	0.0	0.0	0.0	0.0	0.0	0.0	0.0	0.0	0.0	0.0	0.0	0.0	0.0	0.0	0.0
Year 10	0.0	0.0	0.0	0.0	0.0	0.0	0.0	0.0	0.0	0.0	0.0	0.0	0.0	0.0	0.0
Year 15	0.0	0.0	0.0	0.0	0.0	0.0	0.0	0.0	0.0	0.0	0.0	0.0	0.0	0.0	0.0
Year 16	0.0	0.0	0.0	0.0	0.0	0.0	0.0	0.0	0.0	0.0	0.0	0.0	0.0	0.0	0.0
Effectiveness of PCV13 against inpatient all-cause pneumonia (%)															
Year 0	21.6	21.6	14.0	20.9	20.9	13.6	19.6	19.6	12.7	18.4	18.4	12.0	17.2	17.2	11.2
Year 5	21.4	21.4	13.9	20.6	20.6	13.4	19.0	19.0	12.4	17.5	17.5	11.4	15.7	15.7	10.2
Year 10	15.2	15.2	9.9	13.6	13.6	8.8	10.5	10.5	6.8	7.1	7.1	4.6	0.3	0.3	0.2
Year 15	6.4	6.4	4.2	5.4	5.4	3.5	3.5	3.5	2.3	1.1	1.1	0.7	0.0	0.0	0.0
Year 16	5.9	5.9	3.8	4.9	4.9	3.2	3.2	3.2	2.1	1.0	1.0	0.7	0.0	0.0	0.0
Effectiveness of PPSV23 against outpatient all-cause pneumonia (%)															
Year 0	0.0	0.0	0.0	0.0	0.0	0.0	0.0	0.0	0.0	0.0	0.0	0.0	0.0	0.0	0.0
Year 5	0.0	0.0	0.0	0.0	0.0	0.0	0.0	0.0	0.0	0.0	0.0	0.0	0.0	0.0	0.0
Year 10	0.0	0.0	0.0	0.0	0.0	0.0	0.0	0.0	0.0	0.0	0.0	0.0	0.0	0.0	0.0
Year 15	0.0	0.0	0.0	0.0	0.0	0.0	0.0	0.0	0.0	0.0	0.0	0.0	0.0	0.0	0.0
Year 16	0.0	0.0	0.0	0.0	0.0	0.0	0.0	0.0	0.0	0.0	0.0	0.0	0.0	0.0	0.0
Effectiveness of PCV13 against inpatient all-cause pneumonia (%)															
Year 0	21.6	21.6	14.0	20.9	20.9	13.6	19.6	19.6	12.7	18.4	18.4	12.0	17.2	17.2	11.2
Year 5	21.4	21.4	13.9	20.6	20.6	13.4	19.0	19.0	12.4	17.5	17.5	11.4	15.7	15.7	10.2
Year 10	15.2	15.2	9.9	13.6	13.6	8.8	10.5	10.5	6.8	7.1	7.1	4.6	0.3	0.3	0.2
Year 15	6.4	6.4	4.2	5.4	5.4	3.5	3.5	3.5	2.3	1.1	1.1	0.7	0.0	0.0	0.0
Year 16	5.9	5.9	3.8	4.9	4.9	3.2	3.2	3.2	2.1	1.0	1.0	0.7	0.0	0.0	0.0
Medical care costs (per case)															
Bacteremia	$1,886 (R27,156)	$1,886 (R27,156)	$2,219 (R31,948)	$1,932 (R27,827)	$1,932 (R27,827)	$2,265 (R32,619)	$2,076 (R29,887)	$2,505 (R36,069)	$2,838 (R40,862)	$2,551 (R36,740)	$2,788 (R40,143)	$3,027 (R43,594)	$2,884 (R41,533)	$2,884 (R41,533)	$3,264 (R46,996)
Meningitis	$5,024 (R72,340)	$5,024 (R72,340)	$5,200 (R74,880)	$5,024 (R72,340)	$5,024 (R72,340)	$5,200 (R74,880)	$5,141 (R74,034)	$5,141 (R74,034)	$5,322 (R76,634)	$5,444 (R78,387)	$5,444 (R78,387)	$5,683 (R81,834)	$5,683 (R81,834)	$5,683 (R81,834)	$5,683 (R81,834)
Inpatient all-cause pneumonia	$1,649 (R23,745)	$1,746 (R25,135)	$2,032 (R29,257)	$1,649 (R23,745)	$1,889 (R27,196)	$2,078 (R29,928)	$2,221 (R31,988)	$2,365 (R34,049)	$2,697 (R38,842)	$2,647 (R38,123)	$2,934 (R42,244)	$3,410 (R49,097)	$3,123 (R44,976)	$3,123 (R44,976)	$3,599 (R51,829)
Outpatient all-cause pneumonia	$60 (R870)	$60 (R870)	$60 (R870)	$60 (R870)	$60 (R870)	$60 (R870)	$60 (R870)	$60 (R870)	$60 (R870)	$60 (R870)	$60 (R870)	$60 (R870)	$60 (R870)	$60 (R870)	$60 (R870)
Vaccination costs (per vaccination)															
PPSV23	$9.22 (R132.83)	$9.22 (R132.83)	$9.22 (R132.83)	$9.22 (R132.83)	$9.22 (R132.83)	$9.22 (R132.83)	$9.22 (R132.83)	$9.22 (R132.83)	$9.22 (R132.83)	$9.22 (R132.83)	$9.22 (R132.83)	$9.22 (R132.83)	$9.22 (R132.83)	$9.22 (R132.83)	$9.22 (R132.83)
PCV13	$47.59 (R685.27)	$47.59 (R685.27)	$47.59 (R685.27)	$47.59 (R685.27)	$47.59 (R685.27)	$47.59 (R685.27)	$47.59 (R685.27)	$47.59 (R685.27)	$47.59 (R685.27)	$47.59 (R685.27)	$47.59 (R685.27)	$47.59 (R685.27)	$47.59 (R685.27)	$47.59 (R685.27)	$47.59 (R685.27)
Administration costs for PPSV23	$24.96 (R359.42)	$24.96 (R359.42)	$24.96 (R359.42)	$24.96 (R359.42)	$24.96 (R359.42)	$24.96 (R359.42)	$24.96 (R359.42)	$24.96 (R359.42)	$24.96 (R359.42)	$24.96 (R359.42)	$24.96 (R359.42)	$24.96 (R359.42)	$24.96 (R359.42)	$24.96 (R359.42)	$24.96 (R359.42)
Administration costs for PCV13	$2.58 (R37.13)	$2.58 (R37.13)	$2.58 (R37.13)	$2.58 (R37.13)	$2.58 (R37.13)	$2.58 (R37.13)	$2.58 (R37.13)	$2.58 (R37.13)	$2.58 (R37.13)	$2.58 (R37.13)	$2.58 (R37.13)	$2.58 (R37.13)	$2.58 (R37.13)	$2.58 (R37.13)	$2.58 (R37.13)

**Table 5:** Base case model inputs for the HIV-infected public health care sector population.

	18–49 years			50–64 years			65–74 years			75–84 years			85–99 years		
Risk group	Low	Moderate	High	Low	Moderate	Risk group	Low	Moderate	High	Low	Moderate	Risk group	Low	Moderate	High
Population numbers	0	1,725,054	3,027,160	0	198,561	348,438	0	14,302	25,098	0	268	470	0	0	0
Percentage distribution of patients according to disease risk (%)	0	36.3	63.7	0	36.3	63.7	0	36.3	63.7	0	36.3	63.7	0	36.3	63.7
Annual incidence of disease (per 100,000)															
Bacteremia	128.7	128.7	128.7	213.1	213.1	213.1	637.1	637.1	637.1	637.1	637.1	637.1	637.1	637.1	637.1
Meningitis	39.9	39.9	39.9	66.2	66.2	66.2	197.8	197.8	197.8	197.8	197.8	197.8	197.8	197.8	197.8
Inpatient all-cause pneumonia	233.3	233.3	233.3	416.3	416.3	416.3	1396.7	1396.7	1396.7	1549.0	1549.0	1549.0	1599.8	1599.8	1599.8
Outpatient all-cause pneumonia	125.6	125.6	125.6	178.4	178.4	178.4	380.9	380.9	380.9	228.6	228.6	228.6	177.8	177.8	177.8
Annual mortality rate in general population (per 100)	0.6	2.3	2.3	1.6	6.2	6.2	3.1	11.6	11.6	10.0	37.9	37.9	7.4	28.1	28.1
Annual case-fatality rate (per 100)															
Bacteremia	19.4	32.7	32.7	33.6	56.8	56.8	39.9	67.4	67.4	39.9	67.4	67.4	39.9	67.4	67.4
Meningitis	52.1	88.1	88.1	64.2	100.0	100.0	75.0	100.0	100.0	75.0	100.0	100.0	75.0	100.0	100.0
Inpatient all-cause pneumonia	8.6	14.5	14.5	14.8	25.1	25.1	17.6	29.8	29.8	17.6	29.8	29.8	17.6	29.8	29.8
Outpatient all-cause pneumonia	0.0	0.0	0.0	0.0	0.0	0.0	0.0	0.0	0.0	0.0	0.0	0.0	0.0	0.0	0.0
Utilities															
Health-state utility for general population	0.8996	0.8996	0.8245	0.8101	0.8101	0.6897	0.7542	0.7542	0.5688	0.6792	0.6792	0.5206	0.5280	0.5280	0.5071
Disutility due to bacteremia	0.0079	0.0079	0.0079	0.0079	0.0079	0.0079	0.0079	0.0079	0.0079	0.0079	0.0079	0.0079	0.0079	0.0079	0.0079
Disutility due to meningitis	0.0232	0.0232	0.0232	0.0232	0.0232	0.0232	0.0232	0.0232	0.0232	0.0232	0.0232	0.0232	0.0232	0.0232	0.0232
Disutility due to inpatient all-cause pneumonia	0.0060	0.0060	0.0060	0.0060	0.0060	0.0060	0.0060	0.0060	0.0060	0.0060	0.0060	0.0060	0.0060	0.0060	0.0060
Disutility due to outpatient all-cause pneumonia	0.0040	0.0040	0.0040	0.0040	0.0040	0.0040	0.0040	0.0040	0.0040	0.0040	0.0040	0.0040	0.0040	0.0040	0.0040
Effectiveness of PPSV23 against vaccine-type IPD (%)															
Year 0	93.0	93.0	21.0	87.3	87.3	13.7	76.6	76.6	1.4	67.8	67.8	0.0	59.4	59.4	0.0
Year 5	77.1	77.1	18.6	69.0	69.0	12.2	54.1	54.1	0.5	41.3	41.3	0.0	28.8	28.8	0.0
Year 10	29.3	29.3	8.9	22.8	22.8	5.8	12.3	12.3	0.2	4.5	4.5	0.0	0.0	0.0	0.0
Year 15	4.1	4.1	1.8	2.7	2.7	1.2	0.9	0.9	0.0	0.1	0.1	0.0	0.0	0.0	0.0
Year 16	1.0	1.0	0.3	0.7	0.7	0.2	0.2	0.2	0.0	0.0	0.0	0.0	0.0	0.0	0.0
Effectiveness of PCV13 against vaccine-type IPD (%)															
Year 0	84.6	84.6	66.0	82.0	82.0	63.9	76.8	76.8	59.9	72.2	72.2	56.4	67.6	67.6	52.7
Year 5	84.1	84.1	65.6	80.9	80.9	63.1	74.5	74.5	58.1	68.5	68.5	53.5	61.5	61.5	48.0
Year 10	59.6	59.6	46.5	53.3	53.3	41.6	41.0	41.0	32.0	27.7	27.7	21.6	1.1	1.1	0.8
Year 15	25.1	25.1	19.6	21.0	21.0	16.4	13.8	13.8	10.8	4.5	4.5	3.5	0.0	0.0	0.0
Year 16	23.0	23.0	18.0	19.3	19.3	15.0	12.7	12.7	9.9	4.1	4.1	3.2	0.0	0.0	0.0
Effectiveness of PPSV23 against inpatient all-cause pneumonia (%)															
Year 0	0.0	0.0	0.0	0.0	0.0	0.0	0.0	0.0	0.0	0.0	0.0	0.0	0.0	0.0	0.0
Year 5	0.0	0.0	0.0	0.0	0.0	0.0	0.0	0.0	0.0	0.0	0.0	0.0	0.0	0.0	0.0
Year 10	0.0	0.0	0.0	0.0	0.0	0.0	0.0	0.0	0.0	0.0	0.0	0.0	0.0	0.0	0.0
Year 15	0.0	0.0	0.0	0.0	0.0	0.0	0.0	0.0	0.0	0.0	0.0	0.0	0.0	0.0	0.0
Year 16	0.0	0.0	0.0	0.0	0.0	0.0	0.0	0.0	0.0	0.0	0.0	0.0	0.0	0.0	0.0
Effectiveness of PCV13 against inpatient all-cause pneumonia (%)															
Year 0	21.6	21.6	14.0	20.9	20.9	13.6	19.6	19.6	12.7	18.4	18.4	12.0	17.2	17.2	11.2
Year 5	21.4	21.4	13.9	20.6	20.6	13.4	19.0	19.0	12.4	17.5	17.5	11.4	15.7	15.7	10.2
Year 10	15.2	15.2	9.9	13.6	13.6	8.8	10.5	10.5	6.8	7.1	7.1	4.6	0.3	0.3	0.2
Year 15	6.4	6.4	4.2	5.4	5.4	3.5	3.5	3.5	2.3	1.1	1.1	0.7	0.0	0.0	0.0
Year 16	5.9	5.9	3.8	4.9	4.9	3.2	3.2	3.2	2.1	1.0	1.0	0.7	0.0	0.0	0.0
Effectiveness of PPSV23 against outpatient all-cause pneumonia (%)															
Year 0	0.0	0.0	0.0	0.0	0.0	0.0	0.0	0.0	0.0	0.0	0.0	0.0	0.0	0.0	0.0
Year 5	0.0	0.0	0.0	0.0	0.0	0.0	0.0	0.0	0.0	0.0	0.0	0.0	0.0	0.0	0.0
Year 10	0.0	0.0	0.0	0.0	0.0	0.0	0.0	0.0	0.0	0.0	0.0	0.0	0.0	0.0	0.0
Year 15	0.0	0.0	0.0	0.0	0.0	0.0	0.0	0.0	0.0	0.0	0.0	0.0	0.0	0.0	0.0
Year 16	0.0	0.0	0.0	0.0	0.0	0.0	0.0	0.0	0.0	0.0	0.0	0.0	0.0	0.0	0.0
Effectiveness of PCV13 against inpatient all-cause pneumonia (%)															
Year 0	21.6	21.6	14.0	20.9	20.9	13.6	19.6	19.6	12.7	18.4	18.4	12.0	17.2	17.2	11.2
Year 5	21.4	21.4	13.9	20.6	20.6	13.4	19.0	19.0	12.4	17.5	17.5	11.4	15.7	15.7	10.2
Year 10	15.2	15.2	9.9	13.6	13.6	8.8	10.5	10.5	6.8	7.1	7.1	4.6	0.3	0.3	0.2
Year 15	6.4	6.4	4.2	5.4	5.4	3.5	3.5	3.5	2.3	1.1	1.1	0.7	0.0	0.0	0.0
Year 16	5.9	5.9	3.8	4.9	4.9	3.2	3.2	3.2	2.1	1.0	1.0	0.7	0.0	0.0	0.0
Medical care costs (per case)															
Bacteremia	$1,152 (R16,589)	$1,176 (R16,931)	$1,267 (R18,245)	$1,176 (R16,931)	$1,198 (R17,246)	$1,267 (R18,245)	$1,267 (R18,245)	$1,335 (R19,217)	$1,450 (R20,873)	$1,471 (R21,188)	$1,517 (R21,845)	$1,632 (R23,501)	$1,517 (R21,845)	$1,563 (R22,502)	$1,678 (R24,158)
Meningitis	$3,911 (R56,323)	$3,911 (R56,323)	$4,193 (R60,382)	$3,869 (R55,717)	$3,869 (R55,717)	$4,154 (R59,822)	$3,788 (R54,551)	$3,788 (R54,551)	$3,911 (R56,323)	$3,788 (R54,551)	$3,788 (R54,551)	$3,911 (R56,323)	$3,911 (R56,323)	$3,911 (R56,323)	$4,073 (R58,656)
Inpatient all-cause pneumonia	$1,026 (R14,773)	$1,072 (R15,430)	$1,139 (R16,403)	$1,139 (R16,403)	$1,208 (R17,401)	$1,322 (R19,031)	$1,322 (R19,031)	$1,345 (R19,372)	$1,458 (R21,002)	$1,504 (R21,659)	$1,528 (R22,000)	$1,595 (R22,973)	$1,756 (R25,285)	$1,756 (R25,285)	$1,847 (R26,599)
Outpatient all-cause pneumonia	$25 (R356)	$25 (R356)	$25 (R356)	$25 (R356)	$25 (R356)	$25 (R356)	$25 (R356)	$25 (R356)	$25 (R356)	$25 (R356)	$25 (R356)	$25 (R356)	$25 (R356)	$25 (R356)	$25 (R356)
Vaccination costs (per vaccination)															
PPSV23	$7.07 (R101.85)	$7.07 (R101.85)	$7.07 (R101.85)	$7.07 (R101.85)	$7.07 (R101.85)	$7.07 (R101.85)	$7.07 (R101.85)	$7.07 (R101.85)	$7.07 (R101.85)	$7.07 (R101.85)	$7.07 (R101.85)	$7.07 (R101.85)	$7.07 (R101.85)	$7.07 (R101.85)	$7.07 (R101.85)
PCV13	$47.59 (R685.27)	$47.59 (R685.27)	$47.59 (R685.27)	$47.59 (R685.27)	$47.59 (R685.27)	$47.59 (R685.27)	$47.59 (R685.27)	$47.59 (R685.27)	$47.59 (R685.27)	$47.59 (R685.27)	$47.59 (R685.27)	$47.59 (R685.27)	$47.59 (R685.27)	$47.59 (R685.27)	$47.59 (R685.27)
Administration costs for PPSV23	$11.60 (R167.00)	$11.60 (R167.00)	$11.60 (R167.00)	$11.60 (R167.00)	$11.60 (R167.00)	$11.60 (R167.00)	$11.60 (R167.00)	$11.60 (R167.00)	$11.60 (R167.00)	$11.60 (R167.00)	$11.60 (R167.00)	$11.60 (R167.00)	$11.60 (R167.00)	$11.60 (R167.00)	$11.60 (R167.00)
Administration costs for PCV13	$0.00 (R0.00)	$0.00 (R0.00)	$0.00 (R0.00)	$0.00 (R0.00)	$0.00 (R0.00)	$0.00 (R0.00)	$0.00 (R0.00)	$0.00 (R0.00)	$0.00 (R0.00)	$0.00 (R0.00)	$0.00 (R0.00)	$0.00 (R0.00)	$0.00 (R0.00)	$0.00 (R0.00)	$0.00 (R0.00)

The number of persons in the private health care sector per age group was calculated from Council for Medical Schemes data for 2014 [33]. The number of persons in the public health care sector per age group was calculated by subtracting the number of persons in the private health care sector from the total South African population for 2014 [34]. For the private sector HIV population, the HIV prevalence (24 per 1,000 beneficiaries [35]), together with the total population from the Council for Medical Schemes [35], were used to calculate the number of patients in the HIV-infected private sector model. The number of patients in the HIV-infected public sector model was calculated by subtracting the private health care sector HIV-infected population from the total HIV-infected population [36]. The age distributions for both the private and public sector HIV-infected populations were obtained from the Actuarial Society of South Africa (ASSA) model [36].

The annual incidence of bacteremia, meningitis, and all-cause pneumonia per 100,000 persons, assuming no vaccination and no herd effects, by age and risk group, was calculated from Nunes et al. [11] for the established highly active ART (HAART) era. The incidences of meningitis, bacteremia and hospitalized and out-of-hospital all-cause non-bacteremic pneumonia was further split between risk groups using the relative risks (RR) from Kyaw et al. [37] (RR = 8.65 for the moderate-risk group and RR = 44.4 for the high-risk group). For the two HIV models, the incidences were also calculated from Nunes et al. [11], but using the incidences for the HIV-infected only patient group for all risk groups and by not adding any additional risk using the RR from Kyaw et al. [37], as the risk was already for an HIV-infected group. Further to this, the percentage of cases of bacteremia and meningitis that were due to serotypes included in the PCV13 and PPSV23 vaccines, were estimated from data on IPD received from the National Institute for Communicable Disease [12]. It was assumed that these percentages were representative for all four models (private and public health care sector mixed models, and HIV-infected private and public health care sector models).

Indirect (herd) effects were obtained from Delphi panel consensus by asking what the percentage reduction in pneumococcal disease incidence in the adult population is, given the potential herd effects due to the widespread use of PCV13 in young children.

The annual number of all-cause deaths in the general population was obtained from the Statistics South Africa death notification findings [38]. For the HIV models, the rate per 100 persons per age group was multiplied by an additional factor of 3.8 to reflect the higher risk associated with HIV-infected persons compared to HIV-uninfected persons [39]. For the mixed models, only the high-risk group was multiplied with this additional factor.

The CFRs for bacteremia and meningitis were calculated separately [40]. The CFR was calculated by dividing the number of cases with a fatal outcome for each age group by the total number of cases for that age group. It must be noted that the CFR for bacteremia is for bacteremic pneumonia only. Consensus was obtained on this matter during the Delphi panel meeting using the consensus definitions explained above. As there is an additional risk of death for HIV-infected individuals, the CFRs for bacteremia and meningitis for the high-risk group was multiplied by an additional hazard ratio (HR) of 1.69, which is the additional risk of death for HIV-infected individuals compared to HIV-uninfected individuals in the mixed models [40]. For the HIV models, the CFR for each age and risk group was multiplied by this additional HR [40], as all individuals in these models had the higher risk of mortality compared to HIV-negative individuals. No information was found regarding the CFR for persons hospitalized with all-cause non-bacteremic pneumonia in South Africa. Instead, the ratio of the 30 day bacteremic pneumonia mortality (19.5) to the 30 day non-bacteremic pneumonia mortality (5.1) from Lin et al. [41] was used to calculate the non-bacteremic pneumonia CFR from the bacteremic pneumonia CFR. The CFR for outpatient care for all-cause non-bacteremic pneumonia was set to zero, as per the US publication [42]. The same health-state utilities and disutilities as used in the US publication were assumed for all four models, as these values were not available for South Africa [43,44]. A systematic review on health utilities in pneumococcal disease found no studies on health utilities for individuals with pneumococcal disease in sub-Saharan Africa [45]. These utilities and disutilities were used to calculate the QALYs in the model.

For all effectiveness inputs, except for the effectiveness of PCV13 against all-cause NBP, the same effectiveness values as used in the US model were assumed for all four models.

The effectiveness of initial vaccination with PPSV23 against vaccine-type IPD for immunocompetent individuals aged 50 years and older, and for immunocompromised individuals aged 65 years and older, was based on Smith et al. [46]. It was assumed that effectiveness of PPSV23 in immunocompetent individuals aged between 18 and 49 years was the same as for persons aged 50 years [46]. For immunocompromised individuals aged between 18 and 50 years, the initial effectiveness was based on Shapiro et al. [47], while for those aged between 51 and 64 years, effectiveness was estimated by interpolating between values for individuals aged 50 years and 69 years [48]. The rate of decline of protection since vaccination for immunocompromised individuals aged between 18 and 64 years was based on Smith et al. [46]. The effectiveness of vaccination with PPSV23 against all-cause NBP was assumed to be zero. This is based on various published sources and assumptions used in other published economic studies [46,48].

The effectiveness of PCV13 against vaccine-type IPD for immunocompetent individuals was based on the results of the per-protocol population of the CAPiTA study [17]. The per-protocol population was used as this corresponds to immunocompetent individuals throughout the follow-up period. The effectiveness against IPD was anchored on individuals with a mean age of 73 years in the CAPiTA study. Protection was assumed to be stable over the first 5 years of the model, based on the follow-up period in the CAPiTA study (mean of 3.97 years follow-up). The rate of change in the effectiveness of PCV13 for those younger and older than 73 years was equal to 50% of the value for PPSV23. The rate of decline in effectiveness of PCV13 over time (after the initial 5 years) was assumed to be 50% of PPSV23 values. These rates were obtained from an expert panel, including members of the US CDC pneumococcal workgroup. PCV13 effectiveness in immunocompromised individuals was assumed to be 78% of the values for the immunocompetent individuals, based on results from a 9-valent pneumococcal conjugate vaccination in children (with and without HIV) [49]. PPSV23 and PCV13 effectiveness were assumed to be 0% after year 16 of the model time horizon.

The effectiveness of PCV13 against all-cause NBP for immunocompetent individuals was estimated using the effectiveness of PCV13 against vaccine-type non-bacteremic and non-invasive CAP as per the CAPiTA study [17], as well as the percentage of all-cause NBP that is due to serotypes contained in PCV13. To localize the PCV13 effectiveness values for all-cause pneumonia to the South African context, two values were required: the proportion of all-cause pneumonia due to *S*. *pneumoniae* and the serotype coverage for PCV13 from IPD. The proportion of all-cause pneumonia due to *S*. *pneumoniae* was set to 100.0%, as the values provided for mortality and incidence of all-cause pneumonia is only for pneumonia caused by *S*. *pneumoniae*, and not for pneumonia caused by any other pathogens, as the definition requires. The value of serotype coverage for PCV13 from IPD (42.5%) was obtained using the Delphi method consensus. The rate of change with age and rate of decline over the modeling horizon were estimated similarly to that described for the effectiveness of PCV13 against IPD. The initial PCV13 vaccine effectiveness in immunocompromised individuals was assumed to be 65% of the values for immunocompetent individuals, based on Klugman et al. [49]. The waning method was based on absolute levels, and not on the rate of decay. This means that values represent effectiveness in the corresponding year; decline in the interval is estimated via a linear function.

#### Cost data

Unit costs were obtained in South African rand (ZAR), and the model was also run using this currency. Conversion of the currency to US$ (USD) was performed for ease of interpretation and results are shown using both currencies. Conversion was performed using the daily conversion rate from ZAR to USD for 30 November 2015 (where 1 USD = 14.4 ZAR) [50].

Direct medical costs taken into consideration included the cost of the vaccine (PCV13 and PPSV23) and its administration; the cost per event of hospitalization and medication for the treatment of bacteremia, meningitis and all-cause pneumonia; as well as the cost per event of outpatient care for the treatment of all-cause pneumonia. For PPSV23 vaccination, an additional administration fee equal to one consultation fee was added since this vaccine requires a prescription. Microcosting was used to calculate event costs for bacteremia, meningitis and all-cause pneumonia. The resource use per event was based on the literature [51–55] and Delphi panel input. Unit costs for the private sector were sourced from the Department of Health (DoH) Database of Medicine Prices [56] and the National Reference Price Lists (RPL). In the public sector, the Uniform Patient Fee Schedule (UPFS), tender prices and National Health Laboratory Services (NHLS) prices were used [57]. Event costs were obtained by multiplying resource use per event with the unit cost for each element. All costs not in 2015 values were inflated to 2015 values using medical services inflation factors derived from Statistics SA Consumer Price Index publications for the relevant years. Cost details for the private health care sector models are shown in Table 6, while cost details for the public health care sector models are shown in Table 7. Details on resource use and costs for events are shown in S1–S4 Tables.

**Table 6:** Cost details for private health care sector models.

Item	Unit cost (USD) (2015)	Unit cost (ZAR)	Year	Source	Description/code	Tariff/Production cost
Physician consultation	$14.67	R211.30	2009	Medical Practitioners 2009 National Reference Price List	Code 0173 (First hospital consultation/visit)	Tariff
Full blood count	$4.38	R63.00	2009	Medical Practitioners 2009 National Reference Price List	Code 3755 Full blood count (including items 3739, 3762, 3783, 3785, 3791)	Tariff
Blood culture						
*Blood culture*: *Aerobic*	$2.44	R35.10	2009	Medical Practitioners 2009 National Reference Price List	Code 3891 (blood culture: aerobic)	Tariff
*Blood culture*: *Anaerobic*	$2.44	R35.10	2009	Medical Practitioners 2009 National Reference Price List	Code 3892 (blood culture: anaerobic)	Tariff
Urinalysis with culture						
*Urine dipstick*	$0.56	R8.00	2009	Medical Practitioners 2009 National Reference Price List	Code 4188 (urine dipstick)	Tariff
*Miscellaneous (body fluids*, *urine*, *exudate*, *fungi*, *puss*, *scrapings*, *etc*.*)*	$2.06	R29.70	2009	Medical Practitioners 2009 National Reference Price List	Code 3867 (miscellaneous [body fluids, urine, exudate, fungi, puss, scrapings, etc.])	Tariff
*Bacteriological culture*: *Miscellaneous*	$2.63	R37.80	2009	Medical Practitioners 2009 National Reference Price List	Code 3893 (bacteriological culture: miscellaneous)	Tariff
*Viable cell count*	$0.56	R8.09	2009	Medical Practitioners 2009 National Reference Price List	Code 3922 (viable cell count)	Tariff
Serum procalcitonin (PCT)	$19.15	R275.80	2009	Medical Practitioners 2009 National Reference Price List	Code 4539 (procalcitonin: quantitative)	Tariff
Erythrocytic sedimentation rate (ESR)	$1.25	R18.00	2009	Medical Practitioners 2009 National Reference Price List	Code 3743 (Erithrocyte sedimentation rate)	Tariff
Chest X-ray	$19.80	R285.10	2009	Radiology 2009 National Reference Price List	Code 30110 (X-ray of chest two views, PA and lateral)	Tariff
Cerebrospinal fluid (CSF): differential cell count, glucose and protein	$3.19	R45.90	2009	Medical Practitioners 2009 National Reference Price List	Code 4407 (cell count, protein, glucose and chloride)	Tariff
Bacterial culture and sensitivity						
*Bacteriological culture*: *fastidious organisms*	$4.13	R59.40	2009	Medical Practitioners 2009 National Reference Price List	Code 3895 (bacteriological culture: fastidious organisms)	Tariff
*Antibiotic susceptibility test*	$3.33	R47.90	2009	Medical Practitioners 2009 National Reference Price List	Code 3887 (Antibiotic susceptibility test: Per organism)	Tariff
*Rapid automated antibiotic susceptibility*	$7.08	R101.90	2009	Medical Practitioners 2009 National Reference Price List	Code 4653 (Rapid automated antibiotic susceptibility per organism)	Tariff
Serum glucose	$1.51	R21.70	2009	Medical Practitioners 2009 National Reference Price List	Code 4057 (Glucose: Quantitative)	Tariff
Peripheral white cell count	$2.59	R37.30	2009	Medical Practitioners 2009 National Reference Price List	Code 3783 (leucocyte differential count)	Tariff
Lumbar puncture + opening CSF pressure test	$9.94	R143.20	2009	Medical Practitioners 2009 National Reference Price List	Code 2713. Spinal (lumbar) puncture. For diagnosis, for drainage of spinal fluid or for therapeutic indications	Tariff
Sputum gram stain	$2.06	R29.70	2009	Medical Practitioners 2009 National Reference Price List	Code 3867 (Miscellaneous (body fluids, urine, exudate, fungi, puss, scrapings, etc.))	Tariff
Platelet count	$0.94	R13.50	2009	Medical Practitioners 2009 National Reference Price List	Code 3797 (platelet count)	Tariff
Serum electrolytes + urea	$6.60	R95.00	2009	Medical Practitioners 2009 National Reference Price List	Code 4171 (Sodium + potassium + chloride + CO2 + urea)	Tariff
Creatinine	$1.51	R21.70	2009	Medical Practitioners 2009 National Reference Price List	Code 4032 (creatinine)	Tariff
Protein	$1.29	R18.60	2009	Medical Practitioners 2009 National Reference Price List	Code 4117 (protein: total)	Tariff
Albumin	$2.00	R28.80	2009	Medical Practitioners 2009 National Reference Price List	Code 3999 (albumin)	Tariff
Bilirubin						
*Bilirubin*: *Total*	$1.99	R28.60	2009	Medical Practitioners 2009 National Reference Price List	Code 4009 (bilirubin: total)	Tariff
*Bilirubin*: *Conjugated*	$1.51	R21.70	2009	Medical Practitioners 2009 National Reference Price List	Code 4010 (bilirubin: conjugated)	Tariff
Alanine transaminase	$2.25	R32.40	2009	Medical Practitioners 2009 National Reference Price List	Code 4131 (alanine aminotransferase (ALT))	Tariff
High care ward						
*Facility fee*	$203.52	R2 930.70	2009	Private Hospitals 2009 National Reference Price List	Code 215 (High Care Ward, per day)	Tariff
*Specialist fee*	$14.67	R211.30	2009	Medical Practitioners 2009 National Reference Price List	Code 0109 (Hospital follow-up visit to patient in ward or nursing facility)	Tariff
ICU						
*Facility fee*	$317.72	R4 575.20	2009	Private Hospitals 2009 National Reference Price List	Code 201 (intensive care unit, per day)	Tariff
*Specialist fee*	$14.67	R211.30	2009	Medical Practitioners 2009 National Reference Price List	Code 0109 (Hospital follow-up visit to patient in ward or nursing facility)	Tariff
PCV13 vaccine	$47.59	R685.27	2015	Database of Medicine Prices 2015-11-20	Prevenar 13, single exit price, including VAT, per vaccine. NAPPI code 715858001	Single exit price, including VAT
PPSV23 vaccine: Pneumovax	$10.36	R149.21	2015	Database of Medicine Prices 2015-11-20	Pneumovax, single exit price, including VAT, per vaccine. NAPPI code 755826027	Single exit price, including VAT
PPSV23 vaccine: Imovax Pneumo 23	$8.09	R116.44	2015	Database of Medicine Prices 2015-11-20	Imovax Pneumo 23, single exit price, including VAT, per vaccine. NAPPI code 836699009	Single exit price, including VAT
Cost of vaccine administration: PCV13	$1.57	R22.60	2008	Registered Nurses 2008 National Reference Price List	Code 452 (Immunisation)	Tariff
Cost of vaccine administration: PPSV23						
*Immunisation*	$1.57	R22.60	2008	Registered Nurses 2008 National Reference Price List	Code 452 (Immunisation)	Tariff
*Consultation to get script*	$14.67	R211.30	2009	Medical Practitioners 2009 National Reference Price List	Code 0173 (First hospital consultation/visit)	Tariff
Paracetamol	$0.06	R0.80	2015	Database of Medicine Prices 2015-11-20	Average of all 500mg tablets and effervescents with active ingredient paracetamol	Single exit price, including VAT
Ceftriaxone	$0.00	R0.02	2015	Database of Medicine Prices 2015-11-20	Average price per mg of products with active ingredient ceftriaxone only	Single exit price, including VAT
Ampicillin	$0.00	R0.02	2015	Database of Medicine Prices 2015-11-20	Average price per mg of injections with active ingredient ampicillin	Single exit price, including VAT
Vancomycin	$0.01	R0.11	2015	Database of Medicine Prices 2015-11-20	Average price per mg of products with active ingredient vancomycin only	Single exit price, including VAT
Amoxicillin/clavulanate	$3.37	R48.50	2015	Database of Medicine Prices 2015-11-20	Used price of amoxicillin/clavulanic acid IV 1.2g	Single exit price, including VAT
Azithromycin	$0.00	R0.07	2015	Database of Medicine Prices 2015-11-20	Average price per mg of products with active ingredient azithromycin	Single exit price, including VAT
Dexamethasone	$0.31	R4.48	2015	Database of Medicine Prices 2015-11-20	Average price per mg of injections with active ingredient dexamethasone	Single exit price, including VAT
Gentamycin	$0.02	R0.34	2015	Database of Medicine Prices 2015-11-20	Average price per mg of infusions with active ingredient gentamycin/gentamicin only	Single exit price, including VAT
Amoxicillin 3 g per day for 5 days	$0.00	R0.01	2015	Database of Medicine Prices 2015-11-20	Average price per mg of capsules and tablets with active ingredient amoxycillin only	Single exit price, including VAT
Doxycycline	$0.00	R0.02	2015	Database of Medicine Prices 2015-11-20	Average price per mg of products with active ingredient doxycycline	Single exit price, including VAT

USD, United States dollar. ZAR, South African rand.

**Table 7:** Cost details for public health care sector models.

Item	Unit cost (USD) (2015)	Unit cost (ZAR)	Year	Source	Description/code	Tariff/Production cost
Physician consultation						
*Facility fee*	$5.49	R79.00	2015	Uniform Patient Fee Schedule (UPFS) for externally funded patients 2015	Code 1010 (facility fee for outpatient consultation)	Tariff
*GP fee*	$6.11	R88.00	2015	Uniform Patient Fee Schedule (UPFS) for externally funded patients 2015	Code 1011 (GP fee for outpatient consultation)	Tariff
Full blood count	$3.49	R50.25	2012	National Health Laboratory Service (NHLS) State Pricelist 2011/2012	Code 2242 (full blood count incl platelet)	Tariff
Blood culture						
*Blood culture*: *Aerobic*	$3.30	R47.47	2012	National Health Laboratory Service (NHLS) State Pricelist 2011/2012	Code 240 (culture aerobic)	Tariff
*Blood culture*: *Anaerobic*	$2.36	R34.04	2012	National Health Laboratory Service (NHLS) State Pricelist 2011/2012	Code 245 (culture anaerobic)	Tariff
Urinalysis with culture						
*Urine dipstick*	$0.77	R11.11	2012	National Health Laboratory Service (NHLS) State Pricelist 2011/2012	Code 3524 (urine dipstick)	Tariff
*Urine microscopy*	$2.57	R36.95	2012	National Health Laboratory Service (NHLS) State Pricelist 2011/2012	Code 405 (urine microscopy)	Tariff
*Urine culture*	$3.30	R47.47	2012	National Health Laboratory Service (NHLS) State Pricelist 2011/2012	Code 410 (urine culture)	Tariff
*Viable cell count*	$0.72	R10.31	2012	National Health Laboratory Service (NHLS) State Pricelist 2011/2012	Code 421 (viable cell count)	Tariff
Serum procalcitonin (PCT)	$23.37	R336.59	2012	National Health Laboratory Service (NHLS) State Pricelist 2011/2012	Code 3337 (Procalcitonin Quantitative)	Tariff
Erythrocytic sedimentation rate (ESR)	$1.70	R24.54	2012	National Health Laboratory Service (NHLS) State Pricelist 2011/2012	Code 2015 (ESR)	Tariff
Chest X-ray:						
*Facility fee*	$12.15	R175.00	2015	Uniform Patient Fee Schedule (UPFS) for externally funded patients 2015	Code 0520 (facility fee)	Tariff
*GP fee*	$11.67	R168.00	2015	Uniform Patient Fee Schedule (UPFS) for externally funded patients 2015	Code 0521 (Radiology, Cat B—General Medical practitioner)	Tariff
Cerebrospinal fluid (CSF)						
*CSF differential cell count*	$1.81	R26.06	2012	National Health Laboratory Service (NHLS) State Pricelist 2011/2012	Code 210 (CSF cell count)	Tariff
*CSF glucose*	$1.57	R22.59	2012	National Health Laboratory Service (NHLS) State Pricelist 2011/2012	Code 4345 (fluid glucose)	Tariff
*CSF protein*	$1.83	R26.29	2012	National Health Laboratory Service (NHLS) State Pricelist 2011/2012	Code 4235 (CSF/Fluid Protein)	Tariff
Bacterial culture and sensitivity						
*Bacteriological culture*: *fastidious organisms*	$5.18	R74.56	2012	National Health Laboratory Service (NHLS) State Pricelist 2011/2012	Code 585 (culture fastidious organisms)	Tariff
*Sensitivity*	$4.18	R60.20	2012	National Health Laboratory Service (NHLS) State Pricelist 2011/2012	Code 25 (Disc Sensitivity (Per Org))	Tariff
*Rapid automated antibiotic susceptibility*	$8.89	R128.06	2012	National Health Laboratory Service (NHLS) State Pricelist 2011/2012	Code 162 (rapid automated antibiotic susceptibility per organism)	Tariff
Serum glucose	$1.83	R26.29	2012	National Health Laboratory Service (NHLS) State Pricelist 2011/2012	Code 3015 (Glucose)	Tariff
Peripheral white cell count	$1.91	R27.55	2012	National Health Laboratory Service (NHLS) State Pricelist 2011/2012	Code 2000 (differential count)	Tariff
Lumbar puncture + opening CSF pressure test						
*Facility fee*	$26.18	R377.00	2015	Uniform Patient Fee Schedule (UPFS) Code Book 2015—Minor Theatre Procedures	Code 1110 (facility fee, Cat A, level 2 facility)	Tariff
*Specialist fee*	$17.43	R251.00	2015	Uniform Patient Fee Schedule (UPFS) Code Book 2015—Minor Theatre Procedures	Code 1112 (specialist fee, Cat A)	Tariff
Sputum Gram stain	$6.67	R96.05	2012	National Health Laboratory Service (NHLS) State Pricelist 2011/2012	Code 390 (Special ID Gram Pos)	Tariff
Platelet count	$1.29	R18.53	2012	National Health Laboratory Service (NHLS) State Pricelist 2011/2012	Code 2245 (platelet count—manual(1))	Tariff
Serum electrolytes						
*Sodium*	$1.83	R26.29	2012	National Health Laboratory Service (NHLS) State Pricelist 2011/2012	Code 3365 (sodium)	Tariff
*Potassium*	$1.83	R26.29	2012	National Health Laboratory Service (NHLS) State Pricelist 2011/2012	Code 3325 (potassium)	Tariff
*Chloride*	$1.30	R18.76	2012	National Health Laboratory Service (NHLS) State Pricelist 2011/2012	Code 2845 (chloride)	Tariff
*Bicarbonate*	$2.56	R36.82	2012	National Health Laboratory Service (NHLS) State Pricelist 2011/2012	Code 2895 (bicarbonate)	Tariff
Urea	$1.83	R26.29	2012	National Health Laboratory Service (NHLS) State Pricelist 2011/2012	Code 3455 (urea)	Tariff
Creatinine	$1.83	R26.29	2012	National Health Laboratory Service (NHLS) State Pricelist 2011/2012	Code 2960 (creatinine)	Tariff
Protein	$1.57	R22.59	2012	National Health Laboratory Service (NHLS) State Pricelist 2011/2012	Code 3355 (protein total)	Tariff
Albumin	$2.42	R34.86	2012	National Health Laboratory Service (NHLS) State Pricelist 2011/2012	Code 2700 (albumin)	Tariff
Bilirubin						
*Bilirubin direct*	$2.12	R30.57	2012	National Health Laboratory Service (NHLS) State Pricelist 2011/2012	Code 2786 (Bilirubin Direct)	Tariff
*Bilirubin total*	$1.62	R23.28	2012	National Health Laboratory Service (NHLS) State Pricelist 2011/2012	Code 2780 (Bilirubin Total)	Tariff
Alanine transaminase	$2.73	R39.37	2012	National Health Laboratory Service (NHLS) State Pricelist 2011/2012	Code 2685 (Alanine Transaminase)	Tariff
High care ward						
*Facility fee*: *12-hourly rate*	$86.46	R1 245.00	2015	Uniform Patient Fee Schedule (UPFS) for externally funded patients 2015	Code 0620 (facility fee, level 2 facility)	Tariff
*General medical practitioner fee*: *12-hourly rate*	$4.79	R69.00	2015	Uniform Patient Fee Schedule (UPFS) for externally funded patients 2015	Code 0621 (general medical practitioner)	Tariff
ICU						
*Facility fee*: *12-hourly rate*	$227.36	R3 274.00	2015	Uniform Patient Fee Schedule (UPFS) for externally funded patients 2015	Code 0630 (facility fee, level 2 facility)	Tariff
*General medical practitioner fee*: *12-hourly rate*	$5.35	R77.00	2015	Uniform Patient Fee Schedule (UPFS) for externally funded patients 2015	Code 0631 (general medical practitioner)	Tariff
PCV13 vaccine	$47.59	R685.27	2015	Database of Medicine Prices 2015-11-20	Assume the same price as in the private sector (Prevenar 13, single exit price, including VAT, per vaccine. NAPPI code 715858001)	Single exit price, including VAT
PPSV23 vaccine: Pneumovax	$7.07	R101.85	2015	South African Department of Health. Supply and delivery of biological preparations to the Department of Health for the period 1 October 2014 to 30 September 2016 (Contract number HP10-2014BIO)	Item 36. Pneumovax, per vaccine. National stock number 18-971-5733	Tender price, including VAT
Cost of vaccine administration: PPSV23						
*Facility fee*	$5.49	R79.00	2015	Uniform Patient Fee Schedule (UPFS) for externally funded patients 2015	Code 1010 (facility fee for outpatient consultation)	Tariff
*GP fee*	$6.11	R88.00	2015	Uniform Patient Fee Schedule (UPFS) for externally funded patients 2015	Code 1011 (GP fee for outpatient consultation)	Tariff
Paracetamol	$0.01	R0.09	2015	South African Department of Health. Supply and delivery of solid dosage forms and transdermal patches to the Department of Health for the period 1 August 2014 to 31 July 2016 (Contract number HP09-2014SD)	Weighted average of all 500mg tablets and effervescents with active ingredient paracetamol	Tender price, including VAT
Amoxicillin/clavulanate	$0.78	R11.20	2015	South African Department of Health. Supply and delivery of anti-infective medicines (antibiotics, anti-fungal, antiprotozoal and anti-viral agents) to the Department of Health for the period 1 August 2013 to 31 July 2015 (Contract number HP02-2013AI)	Item 22. Weighted average of AMOXYCILLIN 1 000 mg and CLAVULANIC ACID 200 mg Injections, price per injection. National stock number 18-005-7867(VI)	Tender price, including VAT
Azithromycin	$2.52	R36.30	2015	South African Department of Health. Supply and delivery of anti-infective medicines (antibiotics, anti-fungal, antiprotozoal and anti-viral agents) to the Department of Health for the period 1 August 2013 to 31 July 2015 (Contract number HP02-2013AI)	Item 26. Azithromycin 500mg injection. National stock number 18-183-9842(VI)	Tender price, including VAT
Ceftriaxone	$0.00	R0.01	2015	South African Department of Health. Supply and delivery of anti-infective medicines (antibiotics, anti-fungal, antiprotozoal and anti-viral agents) to the Department of Health for the period 1 August 2013 to 31 July 2015 (Contract number HP02-2013AI)	Weighted average price per mg of injections with active ingredient ceftriaxone	Tender price, including VAT
Vancomycin	$0.00	R0.04	2015	South African Department of Health. Supply and delivery of anti-infective medicines (antibiotics, anti-fungal, antiprotozoal and anti-viral agents) to the Department of Health for the period 1 August 2013 to 31 July 2015 (Contract number HP02-2013AI)	Average price per mg of vials with active ingredient vancomycin	Tender price, including VAT
Gentamycin	$0.00	R0.06	2015	South African Department of Health. Supply and delivery of anti-infective medicines (antibiotics, anti-fungal, antiprotozoal and anti-viral agents) to the Department of Health for the period 1 August 2013 to 31 July 2015 (Contract number HP02-2013AI)	Average price per mg of vials with active ingredient gentamicin/gentamycin	Tender price, including VAT
Amoxicillin	$0.00	R0.00	2015	South African Department of Health. Supply and delivery of anti-infective medicines (antibiotics, anti-fungal, antiprotozoal and anti-viral agents) to the Department of Health for the period 1 August 2013 to 31 July 2015 (Contract number HP02-2013AI)	Average price per mg of vials with active ingredient amoxycillin	Tender price, including VAT
Doxycycline	$0.01	R0.20	2015	South African Department of Health. Supply and delivery of anti-infective medicines (antibiotics, anti-fungal, antiprotozoal and anti-viral agents) to the Department of Health for the period 1 August 2013 to 31 July 2015 (Contract number HP02-2013AI)	Average price per 100mg tablet/capsule with active ingredient doxycycline	Tender price, including VAT

USD, United States dollar. ZAR, South African rand.

The human capital approach to calculation of productivity loss costs was used. This considers that productivity loss due to morbidity is represented by lost wages during the period of illness [58]. To calculate the costs related to loss of productivity when an employee was sick, the percentage of persons in the workforce in each age category, the average daily wage for that age category, and the number of work-loss days for each disease, per age and risk group, were calculated. The number of persons in the workforce per age group, as well as the average monthly wage of persons in the workforce, was obtained electronically from Statistics SA (N. Roux, personal email communication, July 9, 2015). Levinsohn et al. [59] indicated that HIV-infected persons were 7.9% more likely to be unemployed, thus for the two HIV models, that percentage was deducted from the percentage in the mixed models.

The number of work-loss days per age group and per disease were assumed to be the same as the number of days required for hospitalization for the disease, plus the additional work-loss days for recovery at home after discharge, as per the Delphi panel inputs. For out-of-hospital non-bacteremic pneumonia cases, the number of work-loss days at home after discharge was used as per the Delphi panel inputs. Only paid employment was considered by multiplying by the percentage of persons in the workforce. As the duration of work-loss due to disease was short (between approximately 5 and 20 days), it is reasonable to consider that there would not be enough time to substitute the sick worker with a new person and to train that person to take over during the absence. However, the number of days would still have a significant impact on productivity and therefore should be considered in the calculation.

## Results

The actual number of adults vaccinated were calculated according to the model input variables. The number of adults vaccinated in the base case are as follows:

- Public sector mixed population PCV13 immunization uptake: 57,957 (0.18%)
- Private sector mixed population PCV13 immunization uptake: 217,930 (3.38%)
- Public sector HIV-infected population PCV13 immunization uptake: 34,157 (0.64%)
- Private sector HIV-infected population PCV13 immunization uptake: 39,421 (25.51%)

### Public health impact

For the mixed scenarios (i.e. including HIV-infected and uninfected patients) in the public and private health care settings, we estimated that vaccination with PCV13 would lead to a reduction of 313 cases (-0.08%) and 716 cases (-0.99%) of IPD, respectively, compared to PPSV23. PCV13 vaccination would also be associated with a reduction in NBP in the public and private health care settings both for patients who are hospitalized and for outpatients (Table 8). Furthermore, PCV13 would lead to a reduction in disease-related deaths (-160 in the public sector [-0.07%] and -354 [-1.17%] in the private sector).

**Table 8:** The public health impact of PCV13 versus PPSV23 vaccination in South African adults.

	*Mixed public health care*			*Mixed private health care*			*HIV+ public health care*			*HIV+ private health care*		
	PCV13	PPSV23	Δ	PCV13	PPSV23	Δ	PCV13	PPSV23	Δ	PCV13	PPSV23	Δ
Number of cases												
IPD	388,973	389,286	-313	71,896	72,612	-716	287,239	287,447	-208	8,079	8,320	-241
NBP												
*Hospitalized*	569,819	570,020	-201	31,116	31,230	-114	434,086	434,212	-126	4,392	4,359	-33
*Outpatient*	256,587	256,688	-101	123,095	123,702	-606	177,901	177,962	-61	13,327	13,512	-185
Number of disease-related deaths	229,526	229,686	-160	29,863	30,216	-354	277,124	277,257	-133	6,153	6,287	-134

Δ, incremental difference (PCV13 –PPSV23); IPD, invasive pneumococcal disease; NBP, non-bacteremic pneumonia.

Amongst the public health care sector adult cohort in which approximately 31 million adults were included in the model, 1.9 million (6.2%) were classified as high-risk patients. The public sector high-risk age distribution was skewed towards a younger population with 12.6% of patients in the 18-49-year age-band, 8.5% in the 50-64-year age-band and only 1.2% in the 85-99-year age-band. This contrasted with the private sector where 6.5 million adults were included in the model, 98,425 (1.52%) were classified as high-risk patients and they were fairly evenly distributed per age-band (1.8% to 4.3%). It is most likely that these demographic differences have led to the differences seen in mortality benefits.

For the HIV-positive model cohorts the results showed a reduction in IPD cases in both the public and private sectors of 208 (-0.07%) and 241 (-2.90%) respectively, with the use of PCV13. There was also a reduction in NBP in both these scenarios for both hospitalized and outpatients (Table 8). Furthermore, the results indicated that PCV13 use would lead to a reduction in disease-related deaths of 133 in the public sector (-0.05%) and 134 (-2.13%) in the private sector.

According to the above results, all strategies considered in the base case indicated that PCV13 would have an improved public health impact compared to PPSV23.

### Cost-effectiveness analysis

The incremental discounted cost-effectiveness results for the four analyses under base case assumptions are shown in Table 9. The undiscounted results are shown in S7 Table in the supporting information. The public sector mixed population shows that while one could expect a discounted saving of approximately $625,000 (R9 million) related to medical care costs over the model lifetime, additional vaccination costs of approximately $1.67 million (R24 million) would be expected with implementation of the PCV13 vaccination strategy. When including all costs related to the vaccination strategy (medical care costs, indirect costs and vaccine costs), the incremental cost of implementing PCV13 compared to PPSV23 vaccination equals approximately $1.001 million (R14.5 million). For this additional investment, the model estimates that 1,402 discounted life years (life years gained LYG) or 1,057 discounted QALYs will be gained. This equates to an ICER of $721 (R10,382)/LYG or $956 (R13,773)/QALY.

**Table 9:** Cost-effectiveness of PCV13 versus PPSV23 vaccination in South African adults in the public and private health care settings for the mixed and HIV-positive populations (discounted).

	*Mixed public health care*			*Mixed private health care*			*HIV+ public health care*			*HIV+ private health care*		
	PCV13	PPSV23	Δ	PCV13	PPSV23	Δ	PCV13	PPSV23	Δ	PCV13	PPSV23	Δ
**Total Costs (thousands of ZAR)**												
Medical Care	10,743,386	10,752,756	-9,371	1,967,545	1,994,238	-26,693	7,684,438	7,690,611	-6,173	226,298	235,068	-8,770
Vaccination	39,716	15,582	24,134	157,432	107,275	50,157	23,407	9,183	14,224	28,477	19,405	9,073
Direct Medical (Medical Care + Vaccination) +Indirect	11,031,926	11,017,372	14,554	2,430,817	2,410,814	20,003	7,821,549	7,813,595	5,080	281,316	282,007	-692
**Total Costs (thousands of USD)**												
Medical Care	746,068	746,719	-651	136,635	138,489	-1,854	533,642	534,070	-429	15,715	16,324	-609
Vaccination	2,758	1,082	1,676	10,933	7,450	3,483	1,625	638	988	1,978	1,348	630
Direct Medical (Medical Care + Vaccination) +Indirect	766,106	765,095	1,011	168,807	167,418	1,389	543,163	542,611	353	19,536	19,584	-48
**Life Years**												
Life Years	473,195,683	473,194,281	1,402	95,358,316	95,355,377	2,939	60,506,664	60,505,689	975	1,759,389	1,758,416	972
Quality-Adjusted Life Years	392,104,506	392,103,449	1,057	78,953,921	78,951,702	2,219	49,026,703	49,025,987	716	1,425,101	1,424,388	713
**ICERs**												
Incremental Cost per Life Year Gained (ZAR) ^a^	-	-	10,382	-	-	6,805	-	-	8,160	-	-	Dominant
Incremental Cost per Quality-Adjusted Life Year Gained (ZAR) ^a^	-	-	13,773	-	-	9,013	-	-	11,106	-	-	Dominant
Incremental Cost per Life Year Gained (USD) ^a^	-	-	721	-	-	473	-	-	567	-	-	Dominant
Incremental Cost per Quality-Adjusted Life Year Gained (USD) ^a^	-	-	956	-	-	626	-	-	771	-	-	Dominant

ICER, Incremental cost-effectiveness ratio; Δ, incremental difference (PCV13 –PPSV23); USD, United States dollar; ZAR, South African rand.

^a^ Costs included in the calculation of the ICER are direct medical costs (medical care + vaccination costs) + indirect costs.

The private sector mixed population shows that while one could expect a discounted saving of approximately $1.8 million (R26 million) related to medical care costs over the model lifetime, additional vaccination costs of approximately $3.47 million (R50 million) would be needed to implement the PCV13 vaccination strategy. When including all costs related to the vaccination strategy (medical care costs, indirect costs and vaccination costs), the incremental cost of implementing PCV13 compared to PPSV23 vaccination equals approximately $1.39 million (R20 million). In return for this additional investment, the model estimates that 2,939 discounted life years or 2,219 QALYs would be gained. This equates to an ICER of $473 (R6,805)/LYG or $626 (R9,013)/QALY.

In the public health care sector HIV-infected cohort, the ICERs are $567 (R8,160)/LYG and $771 (R11,106)/QALY. A similar picture is observed in the private sector HIV-infected cohort, where again the results indicate that PCV13 dominates PPSV23. Dominance means that the new intervention (PCV13) would prove to be more effective and less costly compared to PPSV23. In this scenario, the model indicates that adopting PCV13 would lead to a reduction in total costs of approximately $69,444 (R1 million) yet achieving more life years and QALYs.

When considering only direct medical costs (medical care plus vaccination costs, excluding indirect costs), the following ICERs/QALY were obtained: private sector mixed population $734 (R10,573)/QALY, public sector mixed population $970 (R13,971)/QALY, private sector HIV-infected population $29 (R424)/QALY and public sector HIV-infected population $781 (R11,242)/QALY.

### Sensitivity analysis

Both one-way and probabilistic sensitivity analyses (PSA) were performed to subject the model to uncertainty of the input parameters.

#### One-way sensitivity analysis

**Fig 2:**
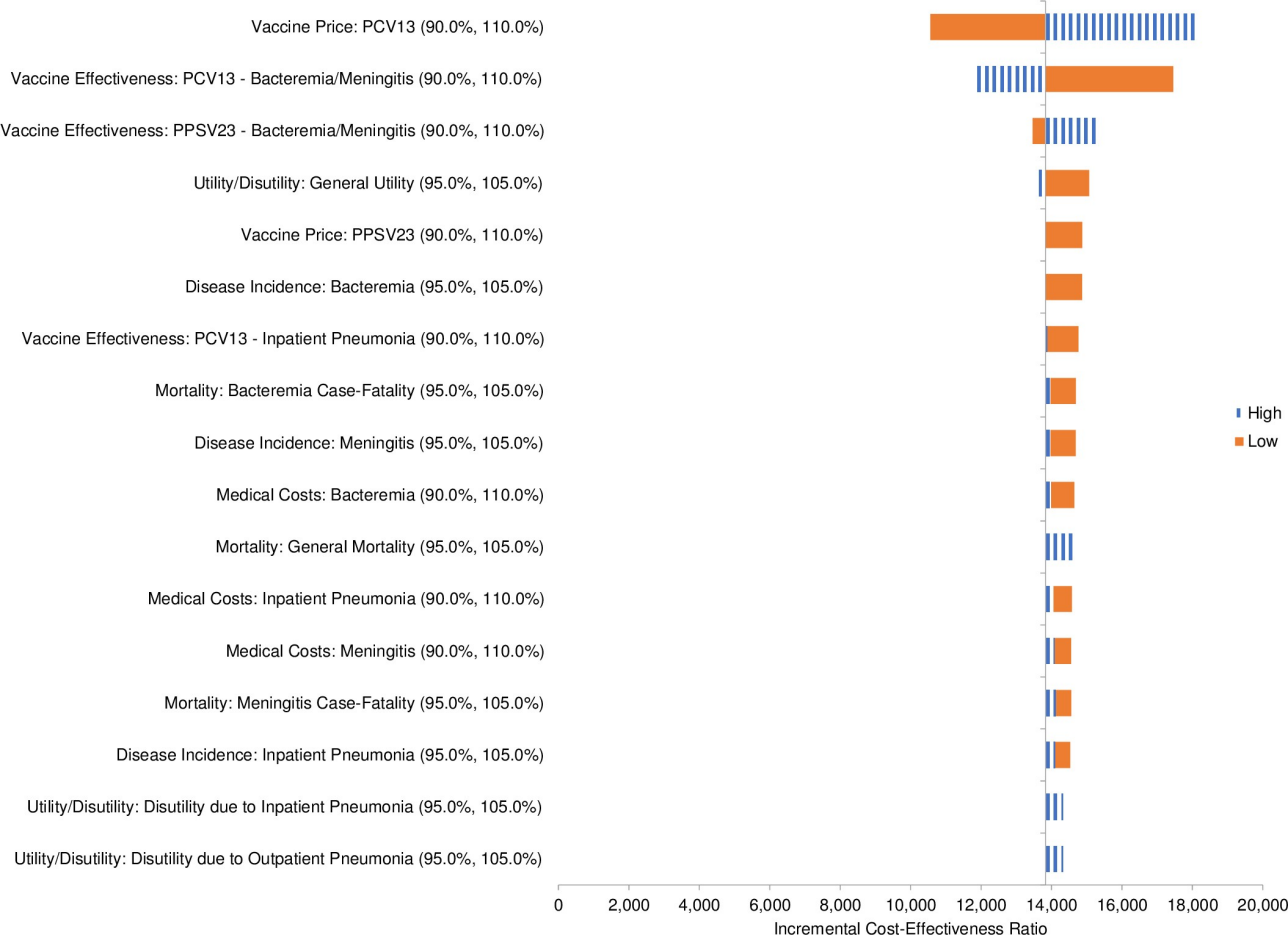
One-way deterministic sensitivity analyses for the public health care sector mixed model. Incremental cost-effectiveness ratio is indicated in 2015 South African rand/QALY.

**Fig 3:**
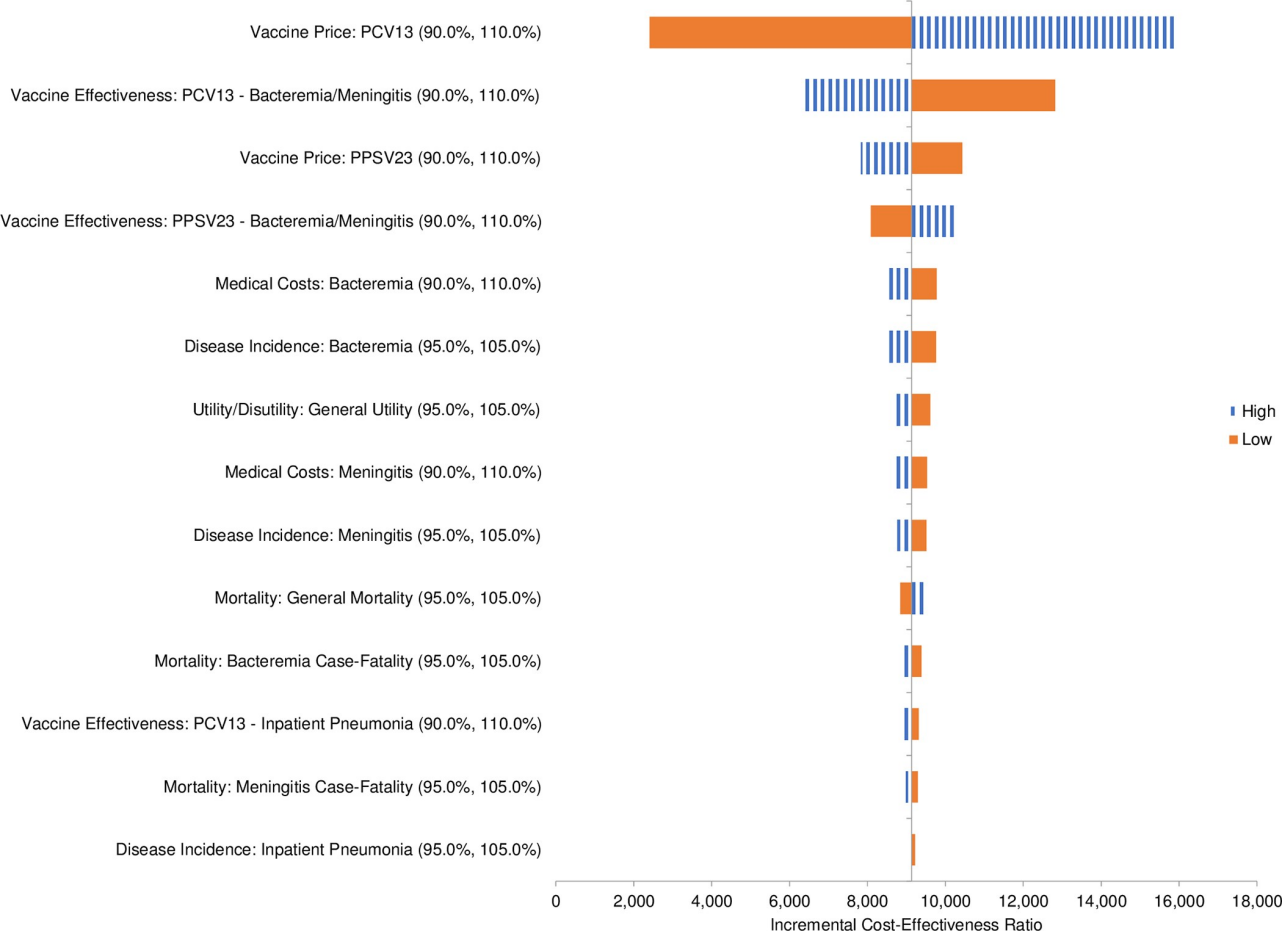
One-way deterministic sensitivity analyses for the private health care sector mixed model. Incremental cost-effectiveness ratio is indicated in 2015 South African rand/QALY.

**Fig 4:**
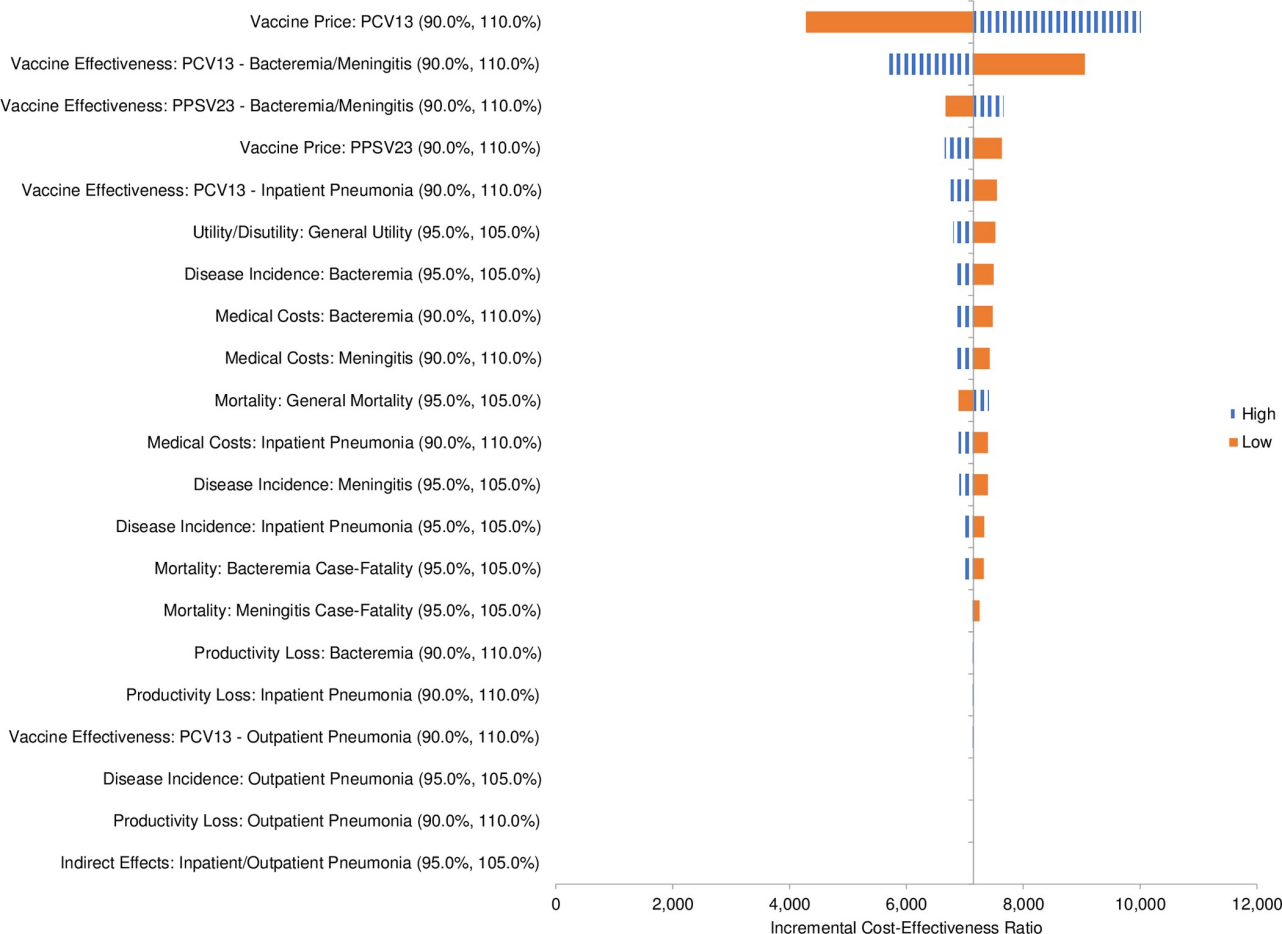
One-way deterministic sensitivity analyses for the public health care sector HIV-infected model. Incremental cost-effectiveness ratio is indicated in 2015 South African rand/QALY.

One-way sensitivity analysis was performed by altering single base case model data inputs to higher or lower values. The results for the one-way sensitivity analyses are shown in Figs 2–4. In the figures, high indicates when a parameter was increased, while low indicates when a parameter was reduced. It shows the deviation from the base case ICER when the parameters are adjusted up or down in the model.

One-way sensitivity analysis showed that vaccine price and vaccine effectiveness were the more sensitive inputs to the model. For the HIV private sector model, all sensitivity scenarios resulted in dominant ICERs, except for vaccine price (high) and vaccine effectiveness (low), which resulted in ICERs of $204/QALY (R2,943/QALY) and $63/QALY (R910/QALY), respectively. Therefore, a graph is not shown for this scenario.

One-way sensitivity analysis is also important where significant assumptions need to be made. This is the case for the utility values used in the model. Using local utility values would be advisable, due to possible differences in social weights between countries [61]. However, these values are not available for South Africa and therefore health-state utilities and disutilities were used from a US model. As can be seen in Figs 2–4, it appears that the model was not sensitive to these parameter assumptions.

The updated vaccine prices as of 22 October 2018 were as follows [62,63]:

- Public sector:
  ○ PCV13: $15.80 (R227.04)
  ○ PPSV23: $9.50 (R136.28)
- Private sector:
  ○ PCV13: $56.30 (R811.23)
  ○ PPSV23: $12.30 (R176.66)

The public sector mixed and HIV-positive models, when populated with the updated prices, resulted in a dominant ICER/LYG and ICER/QALY for PCV13 compared to PPSV23. The private sector mixed model, when populated with the updates prices, resulted in an ICER/LYG of $895 (R12,894) and ICER/QALY of $1,186 (R17,078). The private sector HIV-positive model, when populated with the updated prices, resulted in an ICER/LYG of $182 (R2,619) and ICER/QALY of $248 (R3,571).

According to Statistics South Africa [64], the average life expectancy at birth for 2019 is 61.5 years for males and 67.7 years for females. These values are relevant to the mixed population (HIV-positive and HIV-negative individuals). Sensitivity analysis was performed to reduce the time horizon in the mixed public and private health care sector models to 43 years (61–18 = 43). The following results were obtained when the time horizon was reduced from 82 to 43 years in the mixed public and private health care sector models: mixed public health care sector ICER/QALY of $962 (R13,854) and mixed private health care sector ICER/QALY of $629 (R9,055).

According to Statistics South Africa [64], the average life expectancy at birth for 2019 without HIV/AIDS is 65.6 years for males and 72.7 years for females. Life expectancy according to Johnson et al. [65], for HIV-positive patients on ART, is between 70% and 86% of HIV-negative adults of the same age and sex. Therefore, a life expectancy of approximately 46 years (65.6 x 70% = 46) can be expected for HIV-positive patients on ART. Therefore, a modelling time horizon of approximately 28 years (46–18 = 28). Those not treated with ART are assumed to live approximately 5 to 10 years after infection. The following results were obtained when the time horizon was reduced from 82 to 28 years in the HIV-positive public and private health care sector models: HIV-positive public health care sector ICER/QALY of $813 (R11,711) and HIV-positive private health care sector ICER/QALY remained dominant. Reducing the time horizon to 5 years in the HIV-positive public and private health care sector models resulted in the following: HIV-positive public health care sector ICER/QALY of $8,734 (R125,773) and HIV-positive private health care sector ICER/QALY of $3,504 (R50,460).

#### Probabilistic sensitivity analysis

For the PSA, the following probability distributions were used:

- For incidence rates, vaccine effectiveness and case-fatality rates, a beta distribution was used, with the number of cases and non-cases in the cohort as inputs. This is relevant as the value of these parameters are probabilities ranging between 0 and 1 [66, 67].
- For indirect effects and utility values, a uniform distribution was used, with the minimum and maximum value of these parameters as inputs [66].
- For costs, a log-normal distribution was used, with the mean cost per case and the standard error of the cost per case as inputs. This is relevant, as the value of the costs range from 0 to infinity [66, 67].

**Fig 5:**
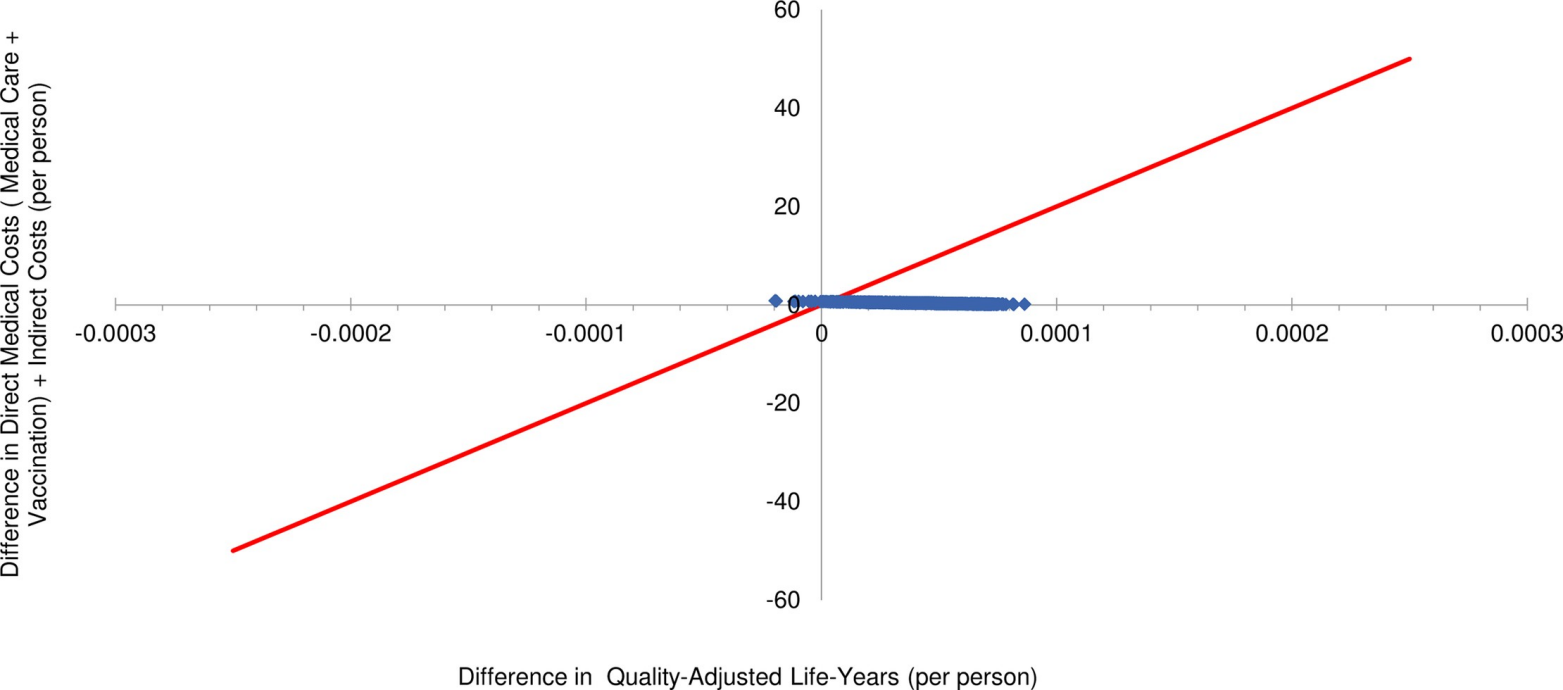
Graphical illustration of PSA for the public health care sector mixed model. Costs are indicated in 2015 South African rand.

**Fig 6:**
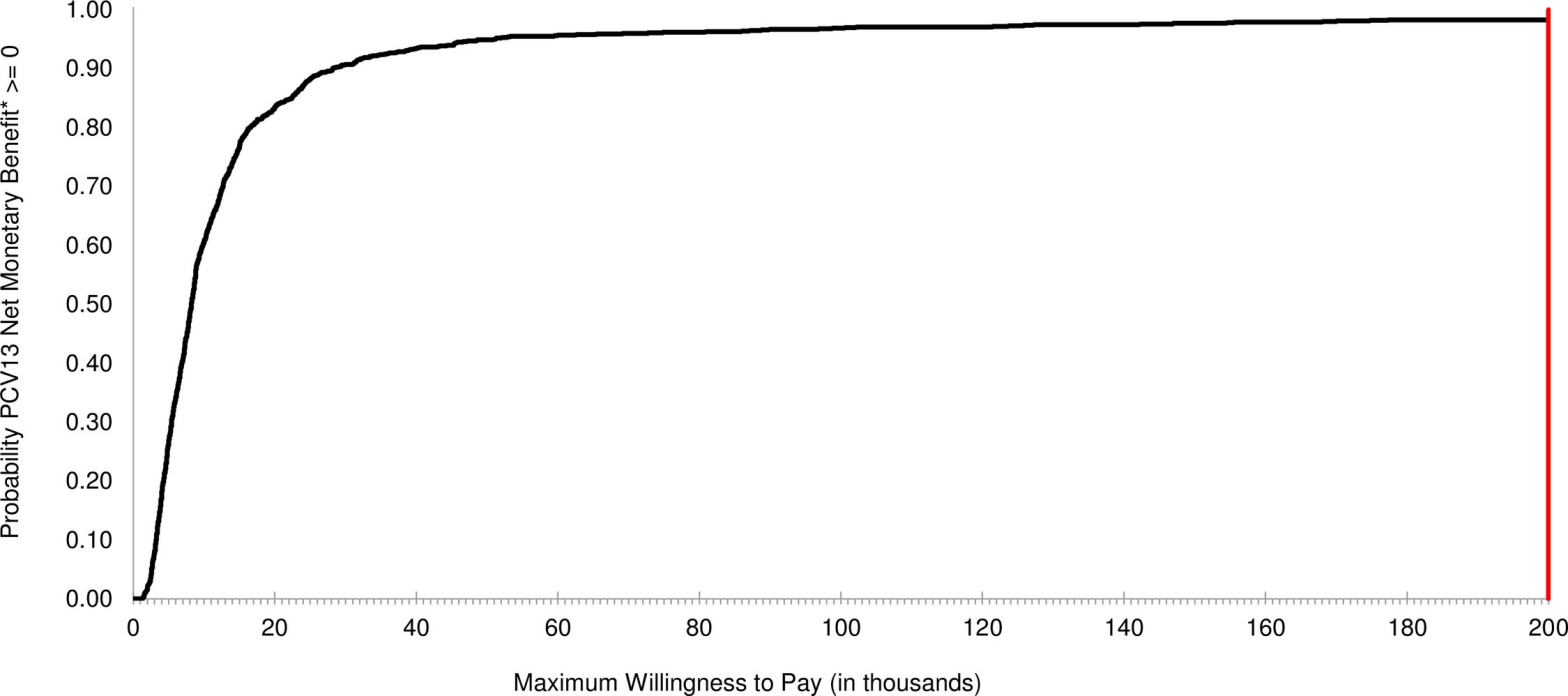
Cost-effectiveness acceptability curve: Public health care sector mixed model. Costs are indicated in 2015 South African rand.

**Fig 7:**
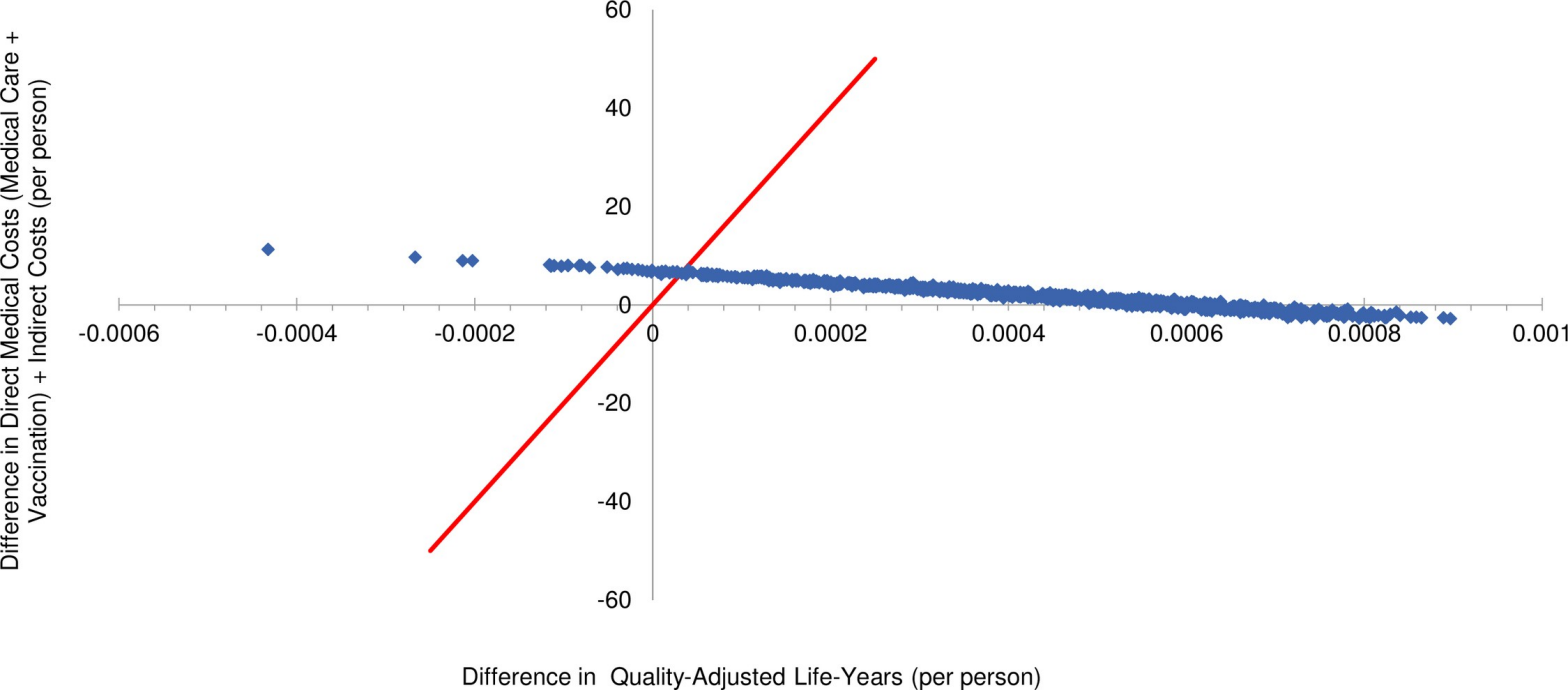
Graphical illustration of PSA for the private health care sector mixed model. Costs are indicated in 2015 South African rand.

**Fig 8:**
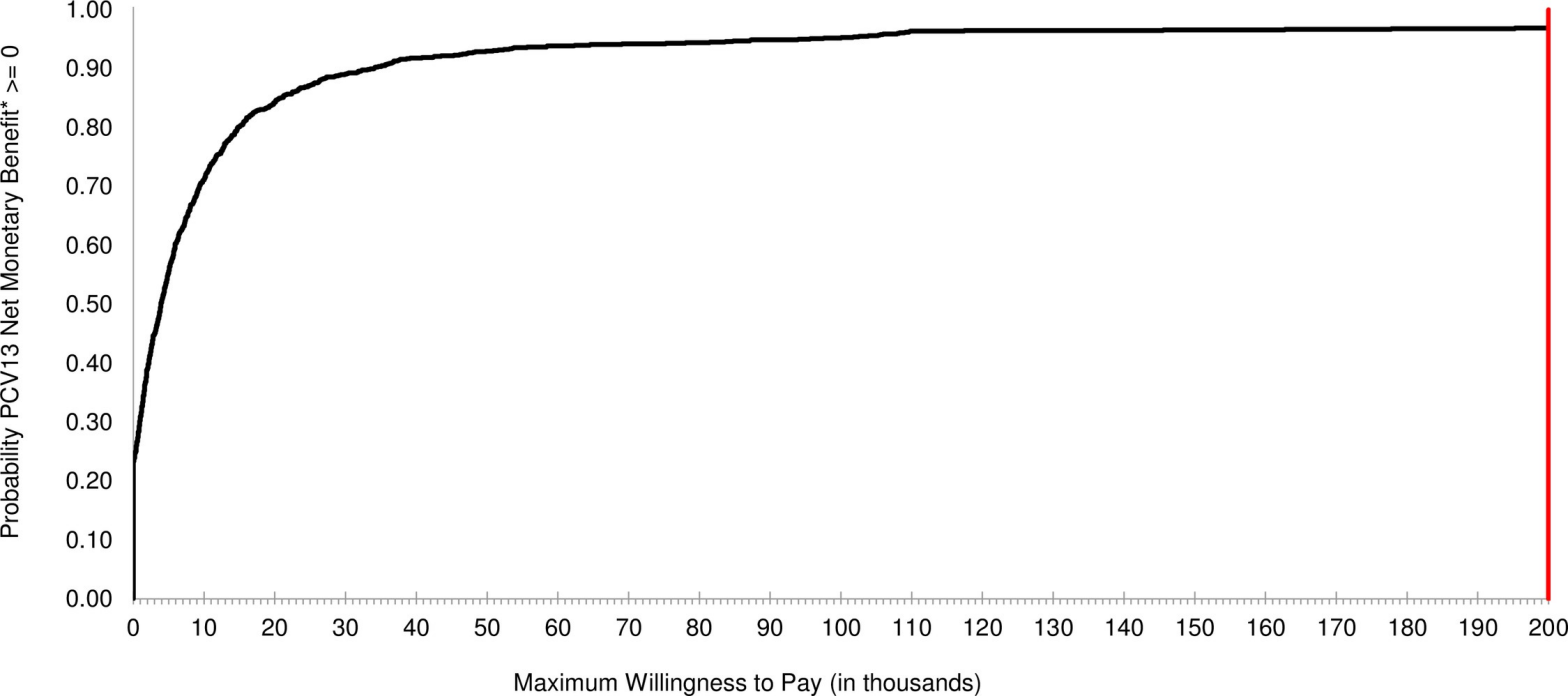
Cost-effectiveness acceptability curve: Private health care sector mixed model. Costs are indicated in 2015 South African rand.

**Fig 9:**
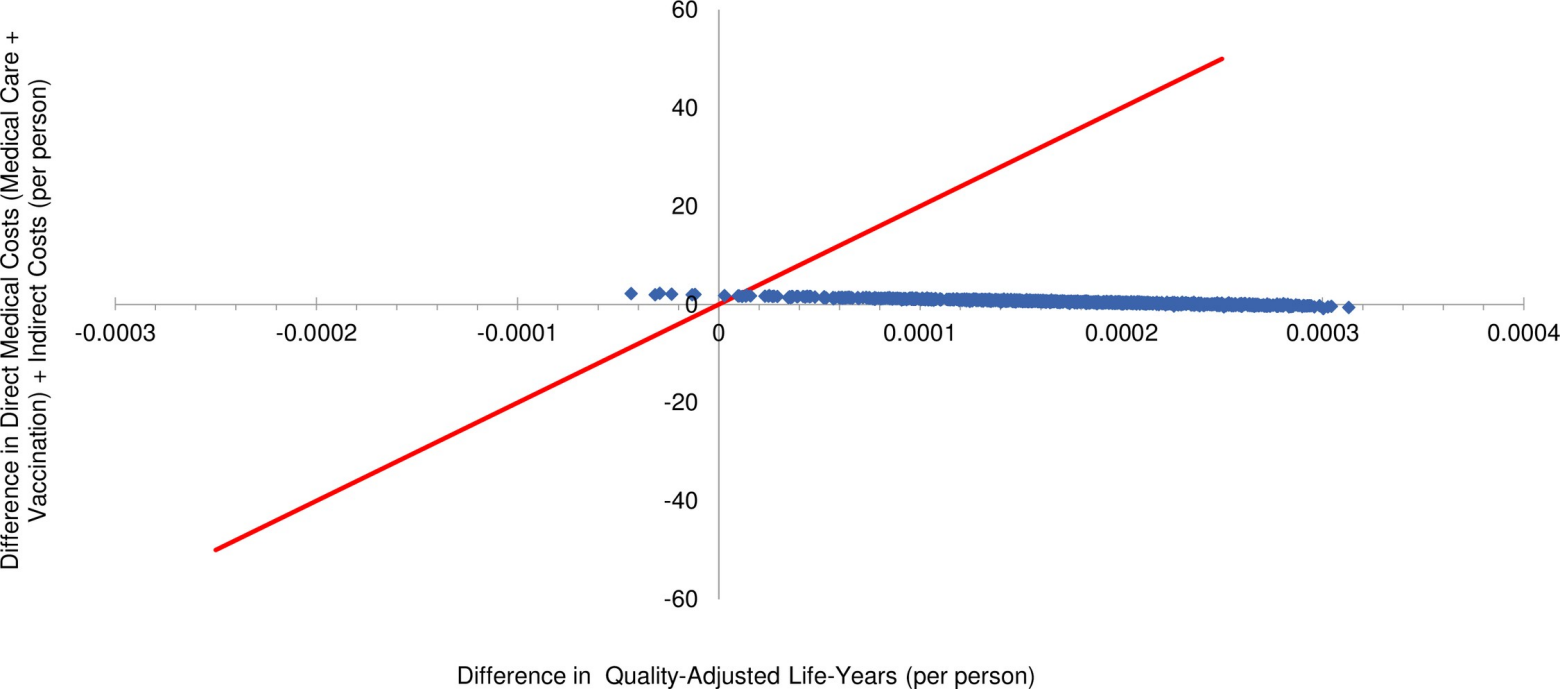
Graphical illustration of PSA for the public health care sector HIV-infected model. Costs are indicated in 2015 South African rand.

**Fig 10:**
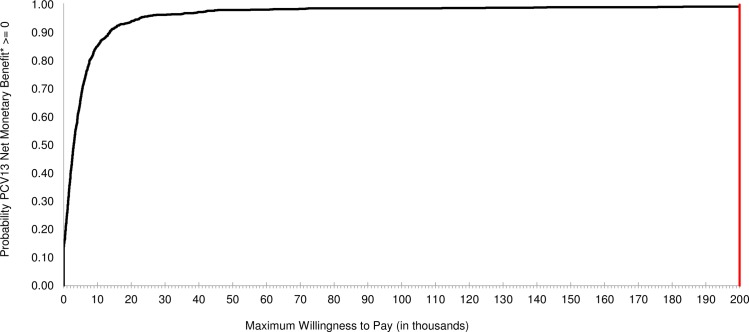
Cost-effectiveness acceptability curve: Public health care sector HIV-infected model. Costs are indicated in 2015 South African rand.

**Fig 11:**
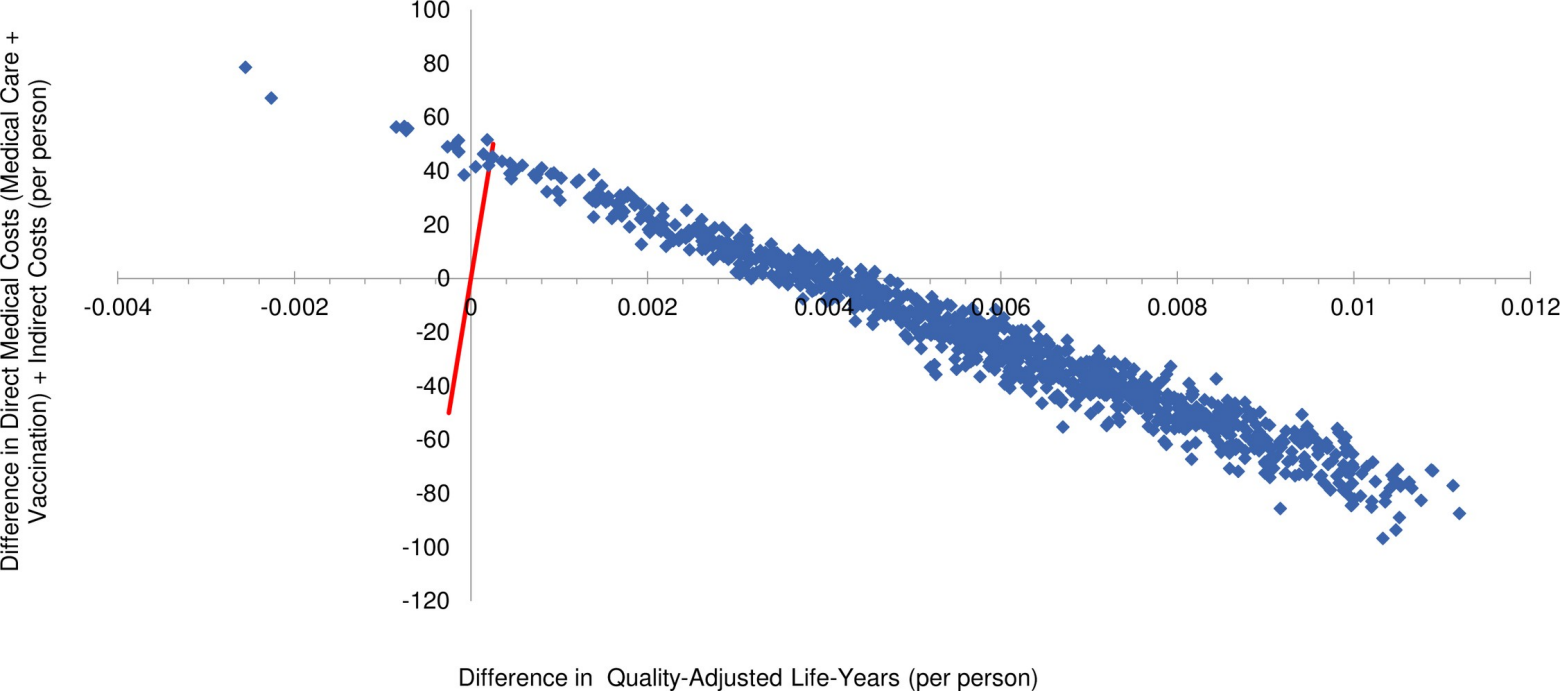
Graphical illustration of PSA for the private health care sector HIV-infected model. Costs are indicated in 2015 South African rand.

**Fig 12:**
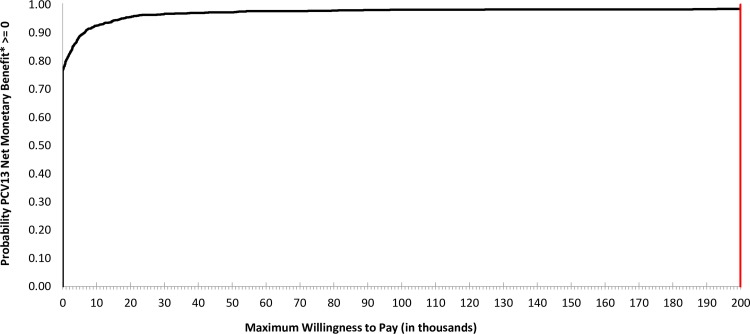
Cost-effectiveness acceptability curve: Private health care sector HIV-infected model. Costs are indicated in 2015 South African rand.

The PSA ICER scatterplots are illustrated in Figs 5, 7, 9 and 11 and show the differences between PCV13 and PPSV23. The quadrant explanation and detailed results for the PSA are provided in the supporting information. The cost-effectiveness acceptability curves are illustrated in Figs 6, 8, 10 and 12, where the net monetary benefit > = 0 means that direct medical costs (medical care costs plus vaccination costs) and indirect costs associated with PCV13 is less than the value of additional benefit achieved.

For the public health care sector mixed model, the PSA and cost-effectiveness acceptability curve indicated that, with a WTP threshold of $6,944 (R100,000), the probability of having a net monetary benefit > = 0 would be 0.963. With a WTP threshold of $13,889 (R200,000) this probability would be 0.972. The ICERs obtained from the PSA indicate that the ICERs are mostly (97%) cost-effective.

For the private health care sector mixed model, the probability of having a net monetary benefit > = 0 was 0.958 at a WTP threshold of $6,944 (R100,000), and 0.969 at a WTP threshold of $13,889 (R200,000). The majority of ICERs obtained from the PSA were cost-effective or dominant (73% below WTP and 24% dominant).

For the public health care sector HIV-infected model, the probability of having a net monetary benefit > = 0 would be 0.980 at a WTP threshold of $6,944 (R100,000), and 0.988 at WTP threshold of $13,889 (R200,000). The results from the PSA indicated that 99% of ICERs obtained were cost-effective or dominant (98% below WTP and 1% dominant) with 1% dominated.

For the private health care sector HIV-infected model, the probability of having a net monetary benefit > = 0 would be 0.973 at a WTP threshold of $6,944 (R100,000), and 0.977 at a WTP threshold of $13,889 (R200,000). The results from the PSA indicated that 98% of ICERs obtained were cost-effective or dominant.

## Discussion

This study was performed to evaluate the cost-effectiveness of immunization of an adult population against pneumococcal disease in South Africa by comparing PPSV23 and PCV13. Four patient cohorts were evaluated. Firstly, given the existence of both a strong private and public health care system in South Africa, the analysis was performed for both these sectors separately. Secondly, given the high prevalence of HIV infection in South Africa, we considered the cost-effectiveness in the two sectors for only the HIV-infected population and secondly for the total South African mixed population (all HIV-infected and uninfected people). These four patient cohorts were then stratified into three groups according to the risk of contracting pneumococcal disease, namely, low-risk, moderate-risk and high-risk patients.

The cost-effectiveness results are based on specific, realistic, vaccination strategies for each of the four patient cohorts and their disease risk characteristics. The base case focus was on vaccinating more of the high- and moderate-risk population and less or none of the low-risk population. Results were then presented based on the incremental population impact of immunizing only a proportion of the stated risk pool. The base case results indicate that the use of PCV13 in favor of PPSV23 is highly cost-effective in both public-sector cohorts with ICERs of $771 (R11,106)/QALY and $956 (R13,773)/QALY for the HIV-infected and mixed population, respectively. The private sector cohort showed similar highly cost-effective results for the mixed population (ICER $626 (R9,013)/QALY) and in the HIV-infected risk pool it showed that PCV13 dominated PPSV23. Even when indirect costs (productivity loss costs) were excluded, all four models show highly cost-effective results.

Before the CAPiTA trial [17], many cost-effectiveness studies were performed based on assumptions and calculations of vaccine effectiveness in adults [68]. Our study used the vaccine effectiveness data based on the CAPiTA trial [17]. Because the CAPiTA trial did not include HIV-infected patients, the PCV13 vaccine efficacy had to be estimated for this cohort of patients. In a study by French et al., PCV7 was effective in preventing recurrent IPD in adult HIV-infected patients [16]. Specifically, the vaccine efficacy against IPD caused by vaccine serotype or serotype-6A was 74% (95% CI: 30%-90%). Smith et al., using an expert panel and Delphi technique, estimated effectiveness of PCV13 in HIV-positive patients for both IPD and NBP [46]. These values are somewhat lower compared to the CAPiTA results for IPD, but almost two times more effective for NBP. In the current study, we opted to keep vaccine effectiveness the same for both the HIV-infected and HIV-uninfected patient cohorts in our base case (based on CAPiTA effectiveness) and subject it to sensitivity analysis.

When comparing our results to other studies in the post-CAPiTA era, the cost-effectiveness study performed by Mangen et al. in the Netherlands [20] closely resembles the South African cost-effectiveness model architecture in terms of age groups, risk stratification and vaccination strategies. Mangen et al., however, compared a single dose of PCV13 to no vaccination and results imply that, in that scenario, PCV13 is cost-effective [20]. Other studies in many other countries show similar results, although the vaccination strategies are diverse [69–72]. It must be noted that these studies are diverse in the population to be vaccinated, vaccination strategies used (sequential use of PCV13->PPSV23, PCV13, revaccination, etc.), comparator (PPSV23 or no vaccine), seroepidemiology and treatment cost. For example, four studies found vaccination with PCV13 to be cost-effective compared to vaccination with PPSV23 [61,70,72,73], and two studies found vaccination with PCV13 to be cost-effective compared to no vaccination [20,72]. In contrast to this, three studies found vaccination with PCV13 in sequence with vaccination with PPSV23 to not be cost-effective compared to PPSV23 alone [69,74,75]. Two of these studies [53,58] reported results for a population of 50 years and older and this might be the reason for the results. In the second study [58], when analyzing patients of 65 and above, the results were cost-effective. The results from the UK study [59] is not surprising given the very low burden of disease related to pneumococcal disease. From the above, it appears that there is a global body of evidence that supports vaccination with PCV13 as a cost-effective intervention when compared to no vaccine or PPSV23. It appears that sequencing PCV13 with PPSV23 is not cost-effective. The present study’s results support the literature, and further differentiates between different risk populations such as HIV-infected patients and mixed populations. This additional information is of particular importance when considering policy implementation strategies in countries like South Africa where there is a high incidence of HIV/AIDS.

As mentioned before, the price of PCV13 remains the most sensitive parameter in the model. In our base case, we took a conservative approach and assumed that the private and public sector price for PCV13 will be the same, but that an administration cost will be incurred in the private sector, but not in the public sector. This conservative approach was followed because a final price for PCV13 for adults was not agreed with the public sector at the time of this publication. However, if one compares the pediatric public sector price for PCV13, it is feasible that one will expect a significantly lower price for PCV13 than what was used in the base case ($18 (R266) vs. $55 (R794) per pediatric dose in the public and private sector, respectively, [60]). When using the pediatric public sector price for PCV13 in our model, both the public sector analyses become dominant. Also, updating the models to the vaccine prices available in October 2018 resulted in both public sector analyses becoming dominant. For both private sector analyses, the ICER/QALY increased, but remained cost-effective.

The model is sensitive to vaccine effectiveness and given the lack of PCV13 vaccine effectiveness data for HIV-infected people, this remains an important shortcoming in our study. However, as was shown in the sensitivity analysis, by decreasing the sensitivity by 10%, vaccination in this group of people remains a cost-effective investment suggesting our results are robust despite the lack of this data.

Vaccine effectiveness was assumed to be stable for the initial 5 years of the modeling horizon (no waning), based on stable vaccine effectiveness during the follow-up period (mean 3.97 years) observed in a post hoc analysis of CAPiTA [76]. As with other studies, another limitation is the fact that no empirical evidence is available after this time period.

The indirect effect of pediatric vaccination in South Africa and its impact on the cost-effectiveness of PCV13 vaccination in adults remains uncertain. The present study’s sensitivity analysis showed, as can be expected, that there is an indirect correlation between the benefits of pediatric vaccination and the ICER. This makes intuitive sense as the more impact the indirect effect has, the lower the burden of pneumococcal disease and therefore the lower the potential impact of adult immunization.

A further limitation of the study is that unpaid lost productivity due to illness, informal health sector costs, such as patient-time costs, unpaid caregiver-time costs and transportation costs, as well as additional non-medical costs, such as future consumption unrelated to health, social services, legal or criminal justice, education, housing and environmental costs were not considered [77].

Probabilistic sensitivity analyses indicated that ICERs were predominantly cost-effective, with only a small percentage of ICERs being dominated or not cost-effective. There is only minor uncertainty regarding the cost-effectiveness of the scenarios at a WTP threshold of $13,889 (R200,000).

The model that was used in this study is a static model and the main limitation (as opposed to a dynamic model) is that the probability of disease exposures is constant over time and unaffected by vaccination. This is probably not realistic if there are high vaccination rates. In the dynamic transmission model, the force of infectivity can change over time due to changes in the size of the susceptible population. A recent systematic review paper [78] of cost-effectiveness analysis publications for adult PCV13 included only static models. The dynamic transmission model requires more data on the transmission aspects and this data is scarce, which leads to many assumptions. This is one of the reasons why most adult pneumococcal vaccine modeling is static. A static model can account for pediatric herd effect.

While it is preferable that local utility values should be considered in economic appraisals, the lack of access to local data required the use of international data. Literature clearly demonstrates statistically significant inter-country differences for health utility values [79]. However, under one-way sensitivity analysis, the use of US-based utility values does not impact on the results from a societal perspective.

The uptake of PCV13 in both the public and private sector is conservative in the model. From a societal perspective, the results are therefore sufficiently robust to provide some guidance to policy makers for consideration and implementation of an immunization strategy for both the public and private sector. It further allows consideration to implement immunization strategies for different patient pools, notably different ages and risk profiles. Given the resource constraints experienced by South Africa, the results of this study provide some insights into the pneumococcal immunization interventions that will provide value for money.

## Supporting information

S1 Table In-hospital costs for bacteremia treatment for the mixed public and private health care sectors. USD, United States dollar; ZAR, South African rand.
(DOCX)

S2 Table In-hospital costs for meningitis treatment for the mixed public and private health care sectors. USD, United States dollar; ZAR, South African rand.
(DOCX)

S3 Table In-hospital cost for all-cause pneumonia treatment for the mixed public and private health care sectors. USD, United States dollar; ZAR, South African rand.
(DOCX)

S4 Table Out-of-hospital costs for all-cause pneumonia treatment for the mixed public and private health care sectors. USD, United States dollar; ZAR, South African rand.
(DOCX)

S5 Table Percentage of persons in workforce and average daily wage per age group for the mixed public and private health care sectors. USD, United States dollar; ZAR, South African rand.
(DOCX)

S6 Table Number of work-loss days for patients in the four sub-cohorts. (DOCX)

S7 Table Cost-effectiveness of PCV13 versus PPSV23 vaccination in South African adults in the public and private health care settings for the mixed and HIV-positive populations (undiscounted). ICER, incremental cost-effectiveness ratio; Δ, incremental difference (PCV13 –PPSV23); USD, United States dollar; ZAR, South African rand. ^a^ Costs included in the calculation of the ICER are direct medical costs (medical care + vaccination costs) + indirect costs.
(DOCX)

S8 Table PSA detailed results—public health care sector mixed model. ICER, incremental cost-effectiveness ratio; WTP, willingness-to-pay; SE, South-East; NE, North-East; SW, South-West; NW, North-West; IC, incremental cost; IE, incremental effectiveness.
(DOCX)

S9 Table PSA detailed results—private health care sector mixed model. ICER, incremental cost-effectiveness ratio; WTP, willingness-to-pay; SE, South-East; NE, North-East; SW, South-West; NW, North-West; IC, incremental cost; IE, incremental effectiveness.
(DOCX)

S10 Table PSA detailed results: Public health care sector HIV-infected model. ICER, incremental cost-effectiveness ratio; WTP, willingness-to-pay; SE, South-East; NE, North-East; SW, South-West; NW, North-West; IC, incremental cost; IE, incremental effectiveness.
(DOCX)

S11 Table PSA detailed results: Private health care sector HIV-infected model. ICER, incremental cost-effectiveness ratio; WTP, willingness-to-pay; SE, South-East; NE, North-East; SW, South-West; NW, North-West; IC, incremental cost; IE, incremental effectiveness.
(DOCX)
