# Supplementary material for: The cost-effectiveness of using pneumococcal conjugate vaccine (PCV13) versus pneumococcal polysaccharide vaccine (PPSV23), in South African adults
Source: PLoS One. 2020 Jan 29;15(1):e0227945. doi: 10.1371/journal.pone.0227945 (PMC6988977; doi:10.1371/journal.pone.0227945)
Supplement: S1 Table — USD, United States dollar; ZAR, South African rand. (DOCX) [file pone.0227945.s001.docx]

**S1 Table. In-hospital costs for bacteremia treatment for the mixed public and private health care sectors.** USD, United States dollar; ZAR, South African rand.

|  | ***Mixed public health care*** | | | | | ***Mixed private health care*** | | | | |
| --- | --- | --- | --- | --- | --- | --- | --- | --- | --- | --- |
| **Input** | **Unit cost (USD 2015)** | **Unit cost (ZAR 2015)** | **Number of units** | **Total cost (USD 2015)** | **Total cost (ZAR 2015)** | **Unit cost (USD 2015)** | **Unit cost (ZAR 2015)** | **Number of units** | **Total cost (USD 2015)** | **Total cost (ZAR 2015)** |
| Physician consultation | 12 | 167 | 10 | 116 | 1,670 | 22 | 322 | 10 | 224 | 3,223 |
| Full blood count | 4 | 60 | 5 | 21 | 302 | 7 | 96 | 5 | 33 | 480 |
| Blood culture | 7 | 98 | 2 | 14 | 196 | 7 | 107 | 2 | 15 | 214 |
| Urinalysis with culture | 9 | 127 | 1 | 9 | 127 | 9 | 129 | 1 | 9 | 129 |
| Serum procalcitonin (PCT) | 28 | 404 | 1 | 28 | 404 | 29 | 421 | 1 | 29 | 421 |
| Erythrocytic sedimentation rate (ESR) | 2 | 29 | 1 | 2 | 29 | 2 | 27 | 1 | 2 | 27 |
| Chest x-ray | 24 | 343 | 1 | 31 | 442 | 30 | 435 | 1 | 40 | 578 |
| Paracetamol: 1000 mg 3 x per day for 5 days |  |  |  | 0 | 3 |  |  |  | 2 | 24 |
| amoxicillin/clavulanate 1.2g IV 3 times a day for 5 days |  |  |  | 12 | 168 |  |  |  | 51 | 728 |
| azithromycin 500 mg once daily for three days |  |  |  | 8 | 109 |  |  |  | 7 | 101 |
| High care ward, number of days according to age (years): |  |  |  |  |  |  |  |  |  |  |
| 18-49 |  |  |  |  |  |  |  |  |  |  |
| Low | 183 | 2,628 | 5 | 913 | 13,140 | 333 | 4,792 | 4 | 1,474 | 21,230 |
| Moderate | 183 | 2,628 | 5 | 936 | 13,482 | 333 | 4,792 | 4 | 1,474 | 21,230 |
| High | 183 | 2,628 | 6 | 1,027 | 14,796 | 333 | 4,792 | 5 | 1,807 | 26,023 |
| 50-64 |  |  |  |  |  |  |  |  |  |  |
| Low | 183 | 2,628 | 5 | 936 | 13,482 | 333 | 4,792 | 5 | 1,521 | 21,901 |
| Moderate | 183 | 2,628 | 5 | 958 | 13,797 | 333 | 4,792 | 5 | 1,521 | 21,901 |
| High | 183 | 2,628 | 6 | 1,027 | 14,796 | 333 | 4,792 | 6 | 1,854 | 26,694 |
| 65-74 |  |  |  |  |  |  |  |  |  |  |
| Low | 183 | 2,628 | 6 | 1,027 | 14,796 | 333 | 4,792 | 5 | 1,664 | 23,962 |
| Moderate | 183 | 2,628 | 6 | 1,095 | 15,768 | 333 | 4,792 | 6 | 2,093 | 30,144 |
| High | 183 | 2,628 | 7 | 1,210 | 17,424 | 333 | 4,792 | 7 | 2,426 | 34,937 |
| 75-84 |  |  |  |  |  |  |  |  |  |  |
| Low | 183 | 2,628 | 7 | 1,232 | 17,739 | 333 | 4,792 | 6 | 2,140 | 30,815 |
| Moderate | 183 | 2,628 | 7 | 1,278 | 18,396 | 333 | 4,792 | 7 | 2,376 | 34,218 |
| High | 183 | 2,628 | 8 | 1,392 | 20,052 | 333 | 4,792 | 8 | 2,616 | 37,668 |
| 85-99 |  |  |  |  |  |  |  |  |  |  |
| Low | 183 | 2,628 | 7 | 1,278 | 18,396 | 333 | 4,792 | 7 | 2,473 | 35,608 |
| Moderate | 183 | 2,628 | 7 | 1,323 | 19,053 | 333 | 4,792 | 7 | 2,473 | 35,608 |
| High | 183 | 2,628 | 8 | 1,438 | 20,709 | 333 | 4,792 | 9 | 2,852 | 41,071 |
