# Supplementary material for: The cost-effectiveness of using pneumococcal conjugate vaccine (PCV13) versus pneumococcal polysaccharide vaccine (PPSV23), in South African adults
Source: PLoS One. 2020 Jan 29;15(1):e0227945. doi: 10.1371/journal.pone.0227945 (PMC6988977; doi:10.1371/journal.pone.0227945)
Supplement: S2 Table — USD, United States dollar; ZAR, South African rand. (DOCX) [file pone.0227945.s002.docx]

**S2 Table. In-hospital costs for meningitis treatment for the mixed public and private health care sectors.** USD, United States dollar; ZAR, South African rand.

|  | ***Mixed public health care*** | | | | | ***Mixed private health care*** | | | | |
| --- | --- | --- | --- | --- | --- | --- | --- | --- | --- | --- |
| **Input** | **Unit cost (USD 2015)** | **Unit cost (ZAR 2015)** | **Number of units** | **Total cost (USD 2015)** | **Total cost (ZAR 2015)** | **Unit cost (USD 2015)** | **Unit cost (ZAR 2015)** | **Number of units** | **Total cost (USD 2015)** | **Total cost (ZAR 2015)** |
| Physician consultation | 12 | 167 | 10 | 116 | 1,670 | 22 | 322 | 10 | 224 | 3,223 |
| Cerebrospinal fluid (CSF) differential cell count + CSF glucose + CSF protein | 6 | 90 | 1 | 6 | 90 | 5 | 70 | 1 | 5 | 70 |
| Bacterial culture and sensitivity | 22 | 315 | 1 | 22 | 315 | 22 | 319 | 1 | 22 | 319 |
| Serum glucose | 2 | 32 | 1 | 2 | 32 | 2 | 33 | 1 | 2 | 33 |
| Peripheral white cell count | 2 | 33 | 1 | 2 | 33 | 4 | 57 | 1 | 4 | 57 |
| Blood culture | 7 | 98 | 1 | 7 | 98 | 7 | 107 | 1 | 7 | 107 |
| Serum procalcitonin (PCT) | 28 | 404 | 1 | 28 | 404 | 29 | 421 | 1 | 29 | 421 |
| Lumbar puncture + opening CSF pressure test | 44 | 628 | 1 | 44 | 628 | 15 | 218 | 1 | 15 | 218 |
| Dexamethasone: 10mg every 6 hours for 4 days |  |  |  | 0 | 5 |  |  |  | 50 | 717 |
| Paracetamol: 1000 mg 3 x / day for 10 days |  |  |  | 20 | 289 |  |  |  | 3 | 48 |
| Ceftriaxone: 4g IV / day for 10 days |  |  |  | 58 | 837 |  |  |  | 67 | 966 |
| Ampicillin: 12g IV / day for 10 days |  |  |  |  |  |  |  |  | 165 | 2,382 |
| Vancomycin: 2g IV / day for 10 days |  |  |  |  |  |  |  |  | 158 | 2,277 |
| High care ward or ICU, number of days according to age band (years): |  |  |  |  |  |  |  |  |  |  |
| 18-49 |  |  |  |  |  |  |  |  |  |  |
| Low | 324 | 4,665 | 11 | 3,606 | 51,921 | 275 | 3,964 | 10 | 4,321 | 62,219 |
| Moderate | 324 | 4,665 | 11 | 3,606 | 51,921 | 275 | 3,964 | 10 | 4,321 | 62,219 |
| High | 324 | 4,665 | 12 | 3,888 | 55,980 | 275 | 3,964 | 11 | 4,497 | 64,759 |
| 50-64 |  |  |  |  |  |  |  |  |  |  |
| Low | 324 | 4,665 | 11 | 3,564 | 51,315 | 275 | 3,964 | 10 | 4,321 | 62,219 |
| Moderate | 324 | 4,665 | 11 | 3,564 | 51,315 | 275 | 3,964 | 10 | 4,321 | 62,219 |
| High | 324 | 4,665 | 12 | 3,849 | 55,420 | 275 | 3,964 | 11 | 4,497 | 64,759 |
| 65-74 |  |  |  |  |  |  |  |  |  |  |
| Low | 324 | 4,665 | 11 | 3,483 | 50,149 | 275 | 3,964 | 11 | 4,438 | 63,912 |
| Moderate | 324 | 4,665 | 11 | 3,483 | 50,149 | 275 | 3,964 | 11 | 4,438 | 63,912 |
| High | 324 | 4,665 | 11 | 3,606 | 51,921 | 275 | 3,964 | 11 | 4,619 | 66,512 |
| 75-84 |  |  |  |  |  |  |  |  |  |  |
| Low | 324 | 4,665 | 11 | 3,483 | 50,149 | 275 | 3,964 | 11 | 4,741 | 68,266 |
| Moderate | 324 | 4,665 | 11 | 3,483 | 50,149 | 275 | 3,964 | 11 | 4,741 | 68,266 |
| High | 324 | 4,665 | 11 | 3,606 | 51,921 | 275 | 3,964 | 12 | 4,980 | 71,712 |
| 85-99 |  |  |  |  |  |  |  |  |  |  |
| Low | 324 | 4,665 | 11 | 3,606 | 51,921 | 275 | 3,964 | 12 | 4,980 | 71,712 |
| Moderate | 324 | 4,665 | 11 | 3,606 | 51,921 | 275 | 3,964 | 12 | 4,980 | 71,712 |
| High | 324 | 4,665 | 12 | 3,768 | 54,254 | 275 | 3,964 | 12 | 4,980 | 71,712 |
