# Supplementary material for: The cost-effectiveness of using pneumococcal conjugate vaccine (PCV13) versus pneumococcal polysaccharide vaccine (PPSV23), in South African adults
Source: PLoS One. 2020 Jan 29;15(1):e0227945. doi: 10.1371/journal.pone.0227945 (PMC6988977; doi:10.1371/journal.pone.0227945)
Supplement: S3 Table — USD, United States dollar; ZAR, South African rand. (DOCX) [file pone.0227945.s003.docx]

**S3 Table. In-hospital cost for all-cause pneumonia treatment for the mixed public and private health care sectors.** USD, United States dollar; ZAR, South African rand.

|  | ***Mixed public health care*** | | | | | ***Mixed private health care*** | | | | |
| --- | --- | --- | --- | --- | --- | --- | --- | --- | --- | --- |
| **Input** | **Unit cost (USD 2015)** | **Unit cost (ZAR 2015)** | **Number of units** | **Total cost (USD 2015)** | **Total cost (ZAR 2015)** | **Unit cost (USD 2015)** | **Unit cost (ZAR 2015)** | **Number of units** | **Total cost (USD 2015)** | **Total cost (ZAR 2015)** |
| Physician consultation | 12 | 167 | 10 | 116 | 1,670 | 22 | 322 | 10 | 224 | 3,223 |
| Chest x-ray | 24 | 343 | 1 | 31 | 442 | 30 | 435 | 1 | 40 | 578 |
| Sputum Gram stain | 8 | 115 | 1 | 8 | 115 | 3 | 45 | 1 | 3 | 45 |
| Blood culture | 7 | 98 | 2 | 14 | 196 | 7 | 107 | 2 | 15 | 214 |
| Full blood count | 4 | 60 | 1 | 4 | 60 | 7 | 96 | 1 | 7 | 96 |
| Platelet count | 2 | 22 | 1 | 2 | 22 | 1 | 21 | 1 | 1 | 21 |
| Serum electrolytes + urea | 11 | 161 | 1 | 11 | 161 | 10 | 145 | 1 | 10 | 145 |
| Creatinine | 2 | 32 | 1 | 2 | 32 | 2 | 33 | 1 | 2 | 33 |
| Protein | 2 | 27 | 1 | 2 | 27 | 2 | 28 | 1 | 2 | 28 |
| Albumin | 3 | 42 | 1 | 3 | 42 | 3 | 44 | 1 | 3 | 44 |
| Bilirubin | 4 | 65 | 1 | 4 | 65 | 5 | 77 | 1 | 5 | 77 |
| Alanine transaminase | 3 | 47 | 1 | 3 | 47 | 3 | 49 | 1 | 3 | 49 |
| Amoxicillin/clavulanate 1.2g IV 3 times a day for 5 days (use Augmentin IV vials) |  |  |  | 12 | 168 |  |  |  | 51 | 728 |
| Gentamycin 320 mg per day for 5 days |  |  |  | 7 | 98 |  |  |  | 33 | 480 |
| Azithromycin 500 mg once daily for three days |  |  |  | 8 | 109 |  |  |  | 7 | 101 |
| High care ward, number of days according to age band: |  |  |  |  |  |  |  |  |  |  |
| 18-49 |  |  |  |  |  |  |  |  |  |  |
| Low | 183 | 2,628 | 4 | 799 | 11,511 | 333 | 4,792 | 4 | 1,235 | 17,780 |
| Moderate | 183 | 2,628 | 5 | 845 | 12,168 | 333 | 4,792 | 4 | 1,331 | 19,170 |
| High | 183 | 2,628 | 5 | 913 | 13,140 | 333 | 4,792 | 5 | 1,617 | 23,291 |
| 50-64 |  |  |  |  |  |  |  |  |  |  |
| Low | 183 | 2,628 | 5 | 913 | 13,140 | 333 | 4,792 | 4 | 1,235 | 17,780 |
| Moderate | 183 | 2,628 | 5 | 982 | 14,139 | 333 | 4,792 | 4 | 1,474 | 21,230 |
| High | 183 | 2,628 | 6 | 1,095 | 15,768 | 333 | 4,792 | 5 | 1,664 | 23,962 |
| 65-74 |  |  |  |  |  |  |  |  |  |  |
| Low | 183 | 2,628 | 6 | 1,095 | 15,768 | 333 | 4,792 | 5 | 1,807 | 26,023 |
| Moderate | 183 | 2,628 | 6 | 1,119 | 16,110 | 333 | 4,792 | 6 | 1,950 | 28,084 |
| High | 183 | 2,628 | 7 | 1,232 | 17,739 | 333 | 4,792 | 7 | 2,283 | 32,876 |
| 75-84 |  |  |  |  |  |  |  |  |  |  |
| Low | 183 | 2,628 | 7 | 1,278 | 18,396 | 333 | 4,792 | 7 | 2,233 | 32,157 |
| Moderate | 183 | 2,628 | 7 | 1,301 | 18,738 | 333 | 4,792 | 8 | 2,519 | 36,279 |
| High | 183 | 2,628 | 8 | 1,369 | 19,710 | 333 | 4,792 | 9 | 2,995 | 43,132 |
| 85-99 |  |  |  |  |  |  |  |  |  |  |
| Low | 183 | 2,628 | 8 | 1,529 | 22,023 | 333 | 4,792 | 8 | 2,709 | 39,010 |
| Moderate | 183 | 2,628 | 8 | 1,529 | 22,023 | 333 | 4,792 | 8 | 2,709 | 39,010 |
| High | 183 | 2,628 | 9 | 1,621 | 23,337 | 333 | 4,792 | 10 | 3,185 | 45,863 |
