# Supplementary material for: The cost-effectiveness of using pneumococcal conjugate vaccine (PCV13) versus pneumococcal polysaccharide vaccine (PPSV23), in South African adults
Source: PLoS One. 2020 Jan 29;15(1):e0227945. doi: 10.1371/journal.pone.0227945 (PMC6988977; doi:10.1371/journal.pone.0227945)
Supplement: S4 Table — USD, United States dollar; ZAR, South African rand. (DOCX) [file pone.0227945.s004.docx]

**S4 Table. Out-of-hospital costs for all-cause pneumonia treatment for the mixed public and private health care sectors.** USD, United States dollar; ZAR, South African rand.

|  | ***Mixed public health care*** | | | | | ***Mixed private health care*** | | | | |
| --- | --- | --- | --- | --- | --- | --- | --- | --- | --- | --- |
| **Input** | **Unit cost (USD 2015)** | **Unit cost (ZAR 2015)** | **Number of units** | **Total cost (USD 2015)** | **Total cost (ZAR 2015)** | **Unit cost (USD 2015)** | **Unit cost (ZAR 2015)** | **Number of units** | **Total cost (USD 2015)** | **Total cost (ZAR 2015)** |
| Physician consultation | 12 | 167 | 2 | 23 | 334 | 22 | 322 | 2 | 45 | 645 |
| Amoxicillin 3 g / day for 5 days |  |  |  | 1 | 11 |  |  |  | 7 | 101 |
| Doxycycline 200 mg stat dose followed by 100 mg twice a day for 5 days |  |  |  | 0 | 2 |  |  |  | 1 | 21 |
