# Supplementary material for: The cost-effectiveness of using pneumococcal conjugate vaccine (PCV13) versus pneumococcal polysaccharide vaccine (PPSV23), in South African adults
Source: PLoS One. 2020 Jan 29;15(1):e0227945. doi: 10.1371/journal.pone.0227945 (PMC6988977; doi:10.1371/journal.pone.0227945)
Supplement: S5 Table — USD, United States dollar; ZAR, South African rand. (DOCX) [file pone.0227945.s005.docx]

**S5 Table. Percentage of persons in workforce and average daily wage per age group for the mixed public and private health care sectors.** USD, United States dollar; ZAR, South African rand.

|  | ***Mixed public health care*** | | | ***Mixed private health care*** | | | ***HIV+ public health care*** | | | ***HIV+ private health care*** | | |
| --- | --- | --- | --- | --- | --- | --- | --- | --- | --- | --- | --- | --- |
| **Age group (years)** | **% of persons in the workforce** | **Average daily wage (USD)** | **Average daily wage (ZAR)** | **% of persons in the workforce** | **Average daily wage (USD)** | **Average daily wage (ZAR)** | **% of persons in the workforce** | **Average daily wage (USD)** | **Average daily wage (ZAR)** | **% of persons in the workforce** | **Average daily wage (USD)** | **Average daily wage (ZAR)** |
| 18-49 | 33 | 8 | 119 | 100 | 23 | 329 | 25 | 8 | 119 | 92 | 23 | 329 |
| 50-64 | 32 | 9 | 136 | 86 | 28 | 399 | 24 | 9 | 136 | 78 | 28 | 399 |
| 65-74 | 6 | 6 | 89 | 24 | 26 | 372 | 0 | 6 | 89 | 16 | 26 | 372 |
| 75-84 | 3 | 8 | 121 | 12 | 24 | 348 | 0 | 8 | 121 | 4 | 24 | 348 |
| 85-99 | 1 | 6 | 81 | 4 | 16 | 229 | 0 | 6 | 81 | 0 | 16 | 229 |
