# Supplementary material for: The cost-effectiveness of using pneumococcal conjugate vaccine (PCV13) versus pneumococcal polysaccharide vaccine (PPSV23), in South African adults
Source: PLoS One. 2020 Jan 29;15(1):e0227945. doi: 10.1371/journal.pone.0227945 (PMC6988977; doi:10.1371/journal.pone.0227945)
Supplement: S6 Table — (DOCX) [file pone.0227945.s006.docx]

**S6 Table. Number of work-loss days for patients in the four sub-cohorts.**

|  | ***Mixed public health care*** | | | | ***Mixed private health care*** | | | |
| --- | --- | --- | --- | --- | --- | --- | --- | --- |
|  | **Hospitalization** | | | **Outpatient care** | **Hospitalization** | | | **Outpatient care** |
| **Age groups (years)** | **Bacteremia** | **Meningitis** | **All-cause pneumonia** | **All-cause pneumonia** | **Bacteremia** | **Meningitis** | **All-cause pneumonia** | **All-cause pneumonia** |
| 18-49 |  |  |  |  |  |  |  |  |
| Low | 11.14 | 19.7 | 9.09 | 5.29 | 10.57 | 18.86 | 8.42 | 5.29 |
| Moderate | 11.27 | 19.7 | 9.34 | 5.29 | 10.57 | 18.86 | 8.71 | 5.29 |
| High | 11.77 | 20.57 | 9.71 | 5.29 | 11.57 | 19.28 | 9.57 | 5.29 |
|  |  |  |  |  |  |  |  |  |
| 50-64 |  |  |  |  |  |  |  |  |
| Low | 11.27 | 19.57 | 9.71 | 5.29 | 10.71 | 18.86 | 8.42 | 5.29 |
| Moderate | 11.39 | 19.57 | 10.09 | 5.29 | 10.71 | 18.86 | 9.14 | 5.29 |
| High | 11.77 | 20.45 | 10.71 | 5.29 | 11.71 | 19.28 | 9.71 | 5.29 |
|  |  |  |  |  |  |  |  |  |
| 65-74 |  |  |  |  |  |  |  |  |
| Low | 11.77 | 19.32 | 10.71 | 5.29 | 11.14 | 19.14 | 10.14 | 5.29 |
| Moderate | 12.14 | 19.32 | 10.84 | 5.29 | 12.43 | 19.14 | 10.57 | 5.29 |
| High | 12.77 | 19.7 | 11.46 | 5.29 | 13.43 | 19.57 | 11.57 | 5.29 |
|  |  |  |  |  |  |  |  |  |
| 75-84 |  |  |  |  |  |  |  |  |
| Low | 12.89 | 19.32 | 11.71 | 5.29 | 12.57 | 19.86 | 11.42 | 5.29 |
| Moderate | 13.14 | 19.32 | 11.84 | 5.29 | 13.28 | 19.86 | 12.28 | 5.29 |
| High | 13.77 | 19.7 | 12.21 | 5.29 | 14 | 20.43 | 13.71 | 5.29 |
|  |  |  |  |  |  |  |  |  |
| 85-99 |  |  |  |  |  |  |  |  |
| Low | 13.14 | 19.7 | 13.09 | 5.29 | 13.57 | 20.43 | 12.85 | 5.29 |
| Moderate | 13.39 | 19.7 | 13.09 | 5.29 | 13.57 | 20.43 | 12.85 | 5.29 |
| High | 14.02 | 20.2 | 13.59 | 5.29 | 14.71 | 20.43 | 14.28 | 5.29 |
