# Supplementary material for: The cost-effectiveness of using pneumococcal conjugate vaccine (PCV13) versus pneumococcal polysaccharide vaccine (PPSV23), in South African adults
Source: PLoS One. 2020 Jan 29;15(1):e0227945. doi: 10.1371/journal.pone.0227945 (PMC6988977; doi:10.1371/journal.pone.0227945)
Supplement: S7 Table — ICER, incremental cost-effectiveness ratio; Δ, incremental difference (PCV13 –PPSV23); USD, United States dollar; ZAR, South African rand. a Costs included in the calculation of the ICER are direct medical costs (medical care + vaccination costs) + indirect costs. (DOCX) [file pone.0227945.s007.docx]

**S7 Table. Cost-effectiveness of PCV13 versus PPSV23 vaccination in South African adults in the public and private health care settings for the mixed and HIV-positive populations (undiscounted).** ICER, incremental cost-effectiveness ratio; Δ, incremental difference (PCV13 – PPSV23); USD, United States dollar; ZAR, South African rand.

^a^ Costs included in the calculation of the ICER are direct medical costs (medical care + vaccination costs) + indirect costs.

|  | ***Mixed public health care*** | | | ***Mixed private health care*** | | | ***HIV+ public health care*** | | | ***HIV+ private health care*** | | |
| --- | --- | --- | --- | --- | --- | --- | --- | --- | --- | --- | --- | --- |
|  | **PCV13** | **PPSV23** | Δ | **PCV13** | **PPSV23** | Δ | **PCV13** | **PPSV23** | Δ | **PCV13** | **PPSV23** | Δ |
| **Total Costs (thousands of ZAR)** | | | | | | | | | | | | |
| Medical Care | 20,922,499 | 20,934,395 | -11,896 | 3,933,613 | 3,967,301 | -33,688 | 16,047,451 | 16,055,258 | -7,807 | 507,162 | 518,353 | -11,191 |
| Vaccination | 39,716 | 15,582 | 24,134 | 157,433 | 107,275 | 50,158 | 23,407 | 9,183 | 14,224 | 28,477 | 19,405 | 9,073 |
| Direct Medical (Medical Care + Vaccination) + Indirect | 21,418,779 | 21,406,807 | 11,973 | 4,643,336 | 4,631,193 | 12,143 | 16,273,407 | 16,267,114 | 6,293 | 585,072 | 588,462 | -3,389 |
| **Total Costs (thousands of USD)** | | | | | | | | | | | | |
| Medical Care | 1,452,951 | 1,453,777 | -826 | 273,168 | 275,507 | -2,339 | 1,114,406 | 1,114,948 | -542 | 35,220 | 35,997 | -777 |
| Vaccination | 2,758 | 1,082 | 1,676 | 10,933 | 7,450 | 3,483 | 1,625 | 638 | 988 | 1,978 | 1,348 | 630 |
| Direct Medical (Medical Care + Vaccination) + Indirect | 1,487,415 | 1,486,584 | 831 | 322,454 | 321,611 | 843 | 1,130,098 | 1,129,661 | 437 | 40,630 | 40,865 | -235 |
| **Life Years** | | | | | | | | | | | | |
| Life Years | 1,059,211,512 | 1,059,208,374 | 3,138 | 211,060,319 | 211,053,973 | 6,346 | 105,312,936 | 105,310,917 | 2,019 | 3,070,003 | 3,067,982 | 2,021 |
| Quality-Adjusted Life Years | 841,701,265 | 841,698,977 | 2,288 | 167,727,470 | 167,722,818 | 4,651 | 83,533,600 | 83,532,146 | 1,454 | 2,433,682 | 2,432,229 | 1,453 |
| **ICERs** | | | | | | | | | | | | |
| Incremental Cost per Life Year Gained (ZAR) ^a^ | - | - | 3,816 | - | - | 1,913 | - | - | 3,117 | - | - | Dominant |
| Incremental Cost per Quality-Adjusted Life Year Gained (ZAR) ^a^ | - | - | 5,232 | - | - | 2,611 | - | - | 4,328 | - | - | Dominant |
| Incremental Cost per Life Year Gained (USD) ^a^ | - | - | 265 | - | - | 133 | - | - | 216 | - | - | Dominant |
| Incremental Cost per Quality-Adjusted Life Year Gained (USD) ^a^ | - | - | 363 | - | - | 181 | - | - | 301 | - | - | Dominant |
