# Supplementary material for: The cost-effectiveness of using pneumococcal conjugate vaccine (PCV13) versus pneumococcal polysaccharide vaccine (PPSV23), in South African adults
Source: PLoS One. 2020 Jan 29;15(1):e0227945. doi: 10.1371/journal.pone.0227945 (PMC6988977; doi:10.1371/journal.pone.0227945)
Supplement: S10 Table — ICER, incremental cost-effectiveness ratio; WTP, willingness-to-pay; SE, South-East; NE, North-East; SW, South-West; NW, North-West; IC, incremental cost; IE, incremental effectiveness. (DOCX) [file pone.0227945.s010.docx]

**S10 Table. PSA detailed results: public health care sector HIV-infected model.** ICER, incremental cost-effectiveness ratio; WTP, willingness-to-pay; SE, South-East; NE, North-East; SW, South-West; NW, North-West; IC, incremental cost; IE, incremental effectiveness.

|  |  |  |  |  | **Simulations** | |
| --- | --- | --- | --- | --- | --- | --- |
| **Quadrant** | **Incremental Cost (IC)** | **Incremental Effectiveness (IE)** | **ICER** | **ICER vs WTP** | **Number** | **Percent** |
| SE | IC < 0 | IE > 0 | Dominant | --- | 6 | 1% |
| NE | IC > 0 | IE > 0 | ICER > 0 | ICER < WTP | 982 | 98% |
| SW | IC < 0 | IE < 0 | ICER > 0 | ICER < WTP | 0 | 0% |
| NE | IC > 0 | IE > 0 | ICER > 0 | ICER > WTP | 3 | 0% |
| SW | IC < 0 | IE < 0 | ICER > 0 | ICER > WTP | 0 | 0% |
| NW | IC > 0 | IE < 0 | Dominated | --- | 9 | 1% |
